# A High Density SNP Array for the Domestic Horse and Extant Perissodactyla: Utility for Association Mapping, Genetic Diversity, and Phylogeny Studies

**DOI:** 10.1371/journal.pgen.1002451

**Published:** 2012-01-12

**Authors:** Molly E. McCue, Danika L. Bannasch, Jessica L. Petersen, Jessica Gurr, Ernie Bailey, Matthew M. Binns, Ottmar Distl, Gérard Guérin, Telhisa Hasegawa, Emmeline W. Hill, Tosso Leeb, Gabriella Lindgren, M. Cecilia T. Penedo, Knut H. Røed, Oliver A. Ryder, June E. Swinburne, Teruaki Tozaki, Stephanie J. Valberg, Mark Vaudin, Kerstin Lindblad-Toh, Claire M. Wade, James R. Mickelson

**Affiliations:** 1 College of Veterinary Medicine, University of Minnesota, St. Paul, Minnesota, United States of America; 2 Department of Population Health and Reproduction, School of Veterinary Medicine, University of California Davis, Davis, California, United States of America; 3 Faculty of Veterinary Science, University of Sydney, Sydney, Australia; 4 Maxwell H. Gluck Equine Research Center, Department of Veterinary Science, University of Kentucky, Lexington, Kentucky, United States of America; 5 Equine Analysis Systems, Midway, Kentucky, United States of America; 6 Institute of Animal Breeding and Genetics, University of Veterinary Medicine Hannover, Hannover, Germany; 7 INRA, UMR 1313, Génétique Animale et Biologie Intégrative, Biologie Intégrative et Génétique Equine, Jouy-en-Josas, France; 8 Equine Research Institute, Japan Racing Association, Utsunomiya, Japan; 9 Animal Genomics Laboratory, School of Agriculture and Food Science, University College Dublin, Dublin, Ireland; 10 Institute of Genetics, Vetsuisse Faculty, University of Bern, Berne, Switzerland; 11 Department of Animal Breeding and Genetics, Swedish University of Agricultural Sciences, Uppsala, Sweden; 12 Veterinary Genetics Laboratory, University of California Davis, Davis, California, United States of America; 13 Department of Basic Sciences and Aquatic Medicine, Norwegian School of Veterinary Science, Oslo, Norway; 14 San Diego Zoo's Institute for Conservation Research, Escondido, California, United States of America; 15 Animal Health Trust, Suffolk, United Kingdom; 16 Department of Molecular Genetics, Laboratory of Racing Chemistry, Utsunomiya, Japan; 17 Science for Life Laboratory, Department of Medical Biochemistry and Microbiology, Uppsala University, Uppsala, Sweden; 18 Broad Institute, Cambridge, Massachusetts, United States of America; University of Liège, Belgium

## Abstract

An equine SNP genotyping array was developed and evaluated on a panel of samples representing 14 domestic horse breeds and 18 evolutionarily related species. More than 54,000 polymorphic SNPs provided an average inter-SNP spacing of ∼43 kb. The mean minor allele frequency across domestic horse breeds was 0.23, and the number of polymorphic SNPs within breeds ranged from 43,287 to 52,085. Genome-wide linkage disequilibrium (LD) in most breeds declined rapidly over the first 50–100 kb and reached background levels within 1–2 Mb. The extent of LD and the level of inbreeding were highest in the Thoroughbred and lowest in the Mongolian and Quarter Horse. Multidimensional scaling (MDS) analyses demonstrated the tight grouping of individuals within most breeds, close proximity of related breeds, and less tight grouping in admixed breeds. The close relationship between the Przewalski's Horse and the domestic horse was demonstrated by pair-wise genetic distance and MDS. Genotyping of other Perissodactyla (zebras, asses, tapirs, and rhinoceros) was variably successful, with call rates and the number of polymorphic loci varying across taxa. Parsimony analysis placed the modern horse as sister taxa to *Equus przewalski*. The utility of the SNP array in genome-wide association was confirmed by mapping the known recessive chestnut coat color locus (*MC1R*) and defining a conserved haplotype of ∼750 kb across all breeds. These results demonstrate the high quality of this SNP genotyping resource, its usefulness in diverse genome analyses of the horse, and potential use in related species.

## Author Summary

We utilized the previously generated horse genome sequence and a large SNP database to design an ∼54,000 SNP assay for use in the domestic horse and related species. The utility of this SNP array was demonstrated through genome-wide linkage disequilibrium, inbreeding and genetic distance measurements within breeds, as well as multidimensional scaling and parsimony analysis. Association mapping confirmed a large conserved segment containing the chestnut coat color locus in domestic horses. We also assess the utility of the SNP array in related species, including the Przewalski's Horse, zebras, asses, tapirs, and rhinoceros. This SNP genotyping tool will facilitate many genetics applications in equids, including identification of genes for health and performance traits, and compelling studies of the origins of the domestic horse, diversity within breeds, and evolutionary relationships among related species.

## Introduction

Horses have held a valued place in human civilization for over 5,000 years through service in war, agriculture, sport and companionship [1]. Over the last several centuries, more than 400 distinct horse breeds have been established by genetic selection for a wide number of desirable phenotypic traits [2]. In contrast to other large domestic animal species including cattle, chickens, sheep, swine, goats and camelids that are selectively bred mainly for production of food (meat, milk, eggs) or fiber, the domestic horse is primarily a utilitarian animal - bred for endurance, strength, speed, and metabolic efficiency [1]. The horses' use as a work animal and means of transport required selection for individuals that were able to perform daily physical activity even when feedstuffs were scarce. The natural athleticism of horses and their enforced intensive exercise regimes makes them outstanding models for study of the musculoskeletal, cardiovascular and respiratory systems, while their natural susceptibility and resistance to infectious agents is useful in studies of the immune system. Understanding the genetic basis for within and among breed variation in equine health, disease and performance traits will continue to provide important information on mammalian biology and genetic mechanisms of disease.

The horse was selected by the National Human Genome Research Institute (NHGRI) for whole genome shotgun sequencing as a representative of the order Perissodactyla. The genome of the female Thoroughbred Twilight was sequenced to 6.8 fold coverage at the Broad Institute of Harvard and MIT accompanied by paired-end sequences from over 150,000 BAC clones performed at the Helmholtz Centre for Infection Research, and the University of Veterinary Medicine Hannover, Germany. This project has produced the EquCab2.0 assembly with a total contig length of 2.43 Gb, 96% of it assigned to chromosomes, and a predicted genome size of 2.67 Gb (http://ncbi.nlm.bih.gov/genome/guide/horse). A significant SNP discovery component within the NHGRI project identified ∼750,000 SNPs from Twilight and ∼400,000 SNPs from seven horses of different breeds, enabling an estimate of the overall frequency of SNPs within the equine genome (∼1/1500 bp), and providing sufficient markers to construct a whole genome SNP panel for use in the domestic horse and related species [3].

This report describes the overall properties and several uses of an equine whole genome SNP array termed the EquineSNP50 BeadChip. Similar to other important domestic animal species, such as the dog, pig, chicken, sheep and cow, this resource has positioned the domestic horse as a viable large animal model for genetic research. Equine researchers are now in an excellent position to evaluate the structure of the genome within and across horse breeds as well as closely related species. Data from this assay will yield important information about selection and population history, and facilitate association mapping studies to allow for the identification of loci associated with both valuable and deleterious traits.

## Results

### SNP array properties, polymorphism, and genome coverage in the domestic horse (*Equus caballus*)

60,000 SNPs from the EquCab2 genome assembly that gave suitable design scores for the Illumina Infinium II assay were selected in an attempt to provide even coverage of the genome. SNPs observed in discovery horses (in reference to the Twilight genome assembly), or in both discovery horses and Twilight, were utilized (Table S1). Of the 354 horses from 14 different breeds selected for genotyping, 3 individuals failed to genotype (Table S2). Analysis of 8 pairs of replicate samples (Twilight and the seven SNP discovery horses) resulted in perfect replication of 868,820 genotypes (replication frequency of 1.0). Mendelian inheritance was confirmed in 15 of 18 trios (Table S3).

54,602 SNPs provided genotype data, and 53,524 SNPs were validated (defined as having at least one heterozygous genotype call), indicating overall assay conversion and validation rates of 0.910 and 0.980, respectively. The validation rates were highest for SNPs that were observed in a single discovery breed (0.990) when compared to Twilight, or observed in any two discovery breeds (0.989) (Table S1). 53,066 of the validated SNPs (99.1%) were polymorphic (defined as minor allele frequency (MAF>0.01) in the entire sample set. The average spacing between functional SNPs on the 31 autosomes was 43.1 kb. There were 12 gaps greater than 500 kb across the 31 autosomes, with the largest gap being 1,647.5 kb on ECA6 (Table S4). Coverage on ECAX (average inter-SNP spacing of 48.88 kb) was lower than the rest of the genome (Table S4).

The number of polymorphic SNPs (MAF≥0.01) within a breed ranged from 43,287 to 52,085 (79% to 95%); 26,473 (48.5%) SNPs were polymorphic in every breed (Table S5). 90% of informative SNPs (MAF>0.05 across breeds) were less than 110 kb apart, and 95% of informative SNPs were less than 150 kb apart (Figure S1). The discovery breed source of the SNPs did not greatly affect their informativeness (MAF>0.05) in the 14 analyzed breeds individually, or as a whole (Table S6).

### Utility of the EquineSNP50 BeadChip in extant Perissodactyla

Genotyping was attempted in 53 individuals from 18 species evolutionarily related to the domestic horse (Table S7). The extant Perissodactyla (odd-toed hoofed mammals) include three families: the Equidae (horses, asses and zebras), the Rhinocerotidae (rhinos), and the Tapiridae (tapirs), divided into two suborders, the Hippomorpha (horses, asses and zebras) and the Ceratomorpha (rhinos and tapirs) [4]. Of the 53 individuals genotyped, one single zebra (*Equus zebra hartmannae*) completely failed to genotype. Individual genotyping rates were slightly lower across the Hippomorpha (mean 0.959), and dramatically lower across the Ceratomorpha (mean 0.246) when compared to the domestic horse (mean 0.996) (Table S7). Quality scores (GC10, see Materials and Methods) in the Hippomorpha (mean = 0.705) were similar to those in the domestic horse (mean = 0.730), however mean GC10 scores were much lower in Ceratomorpha (mean = 0.236) (Table S7).

Due to lower quality scores and lower genotyping rates in some species, the genotypes in the Perissodactyla were further filtered based on raw intensity scores, individual genotyping rates, and SNP genotyping rates (see Materials and Methods and Text S1 for details). The number of loci called after filtering for signal intensity and genotyping rates are presented in Table S8. In the Hippomorpha our filtering criteria had little impact on genotyping rates, decreasing the call rate by only 1 to 3%. In contrast, filtering criteria decreased the call rates by 45 to 57% in the Ceratomorpha, suggesting that a large portion of the initial genotyping calls were unreliable. Further, mean GC10 scores for the remaining SNPs after filtering were 0.721 across all Hippomorpha and 0.389 across all Ceratomorpha respectively (Table S8), suggesting that data quality in the Ceratomorpha was questionable even after additional filtering. Thus only the Hippomorpha data were analyzed further.

In the Hippomorpha, conversion rates ranged from 0.891 in the Przewalski's Horse (*Equus przewalskii*) to 0.834 in the Hartmann's Mountain Zebra (*Equus zebra hartmannae*). The number of validated loci ranged from 265 (0.8%) in the Somali Wild Ass (*Equus asinus somalicus*) to 26,859 (50.8%) in the Przewalski's Horse (Table S8). The average observed heterozygosity in the Hippomorpha (excluding the domestic horse) ranged from 0.003 in the Domestic Ass, Somali Wild Ass, Grevy's zebra and Hartmann's Mountain zebra to 0.168 in the Przewalski's Horse (Table S8). Mean MAF in the nine Przewalski's Horses was 0.126.

### Minor allele frequency and genetic diversity within and across domestic horse breeds

The number of informative SNPs within breeds ranged from 37,053 (68%) in the Norwegian Fjord to 47,669 (87%) in the Quarter Horse (Table S5). Mean MAF within breeds also ranged from 0.180 to 0.232 in the Norwegian Fjords and Quarter Horses, respectively. 17,428 SNPs were informative (MAF≥0.05) in every breed and 49,603 SNPs were informative within the entire sample set (across all breeds). The overall MAF across all breeds was 0.236 (SD = 0.139), and the median MAF was 0.224. The Mongolian breed displayed the highest genetic diversity, H_E_ = 0.292, whereas genetic diversity was the lowest in the Thoroughbred H_E_ = 0.247 (Table S5).

### Genome-wide linkage disequilibrium analysis within and across domestic horse breeds

**Figure 1:**
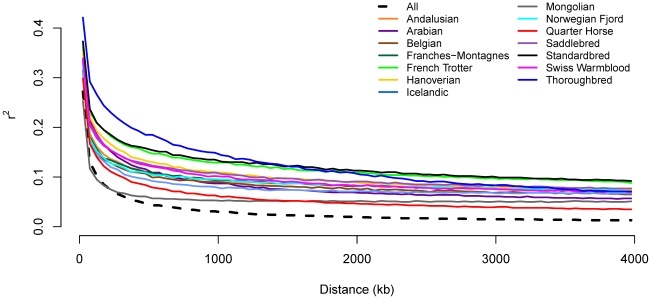
Decline in genome-wide linkage disequilibrium across and within breeds. Genome-wide linkage disequilibrium (LD) was estimated both within a given breed, and across all breeds, by calculating *r^2^* values between all pairs of SNPs with inter-SNP distances of less than 4 Mb as described in Materials and Methods.

Genotypes for all SNP pairs less than 4 Mb apart were evaluated to estimate genome-wide linkage disequilibrium (LD) (as *r^2^*) within and across breeds. As expected, LD was higher within a breed than across breeds. Initial LD declined rapidly across all horses with mean *r^2^* dropping below 0.2 by 50 kb (Figure 1 and Figure S2). Within breed *r^2^* values dropped most rapidly in the Mongolian, however, *r^2^* was below 0.2 within 100 to 150 kb in the majority of breeds. LD was initially highest in the Thoroughbred, where *r^2^* does not drop below 0.2 until 400 kb, and remained higher than other breeds until approximately 1,200 kb. The extent of long-range LD was the highest in the Standardbred and French Trotter (Figure 1).

### Inbreeding, genetic distance, and relationships between domestic horse breeds

**Figure 2:**
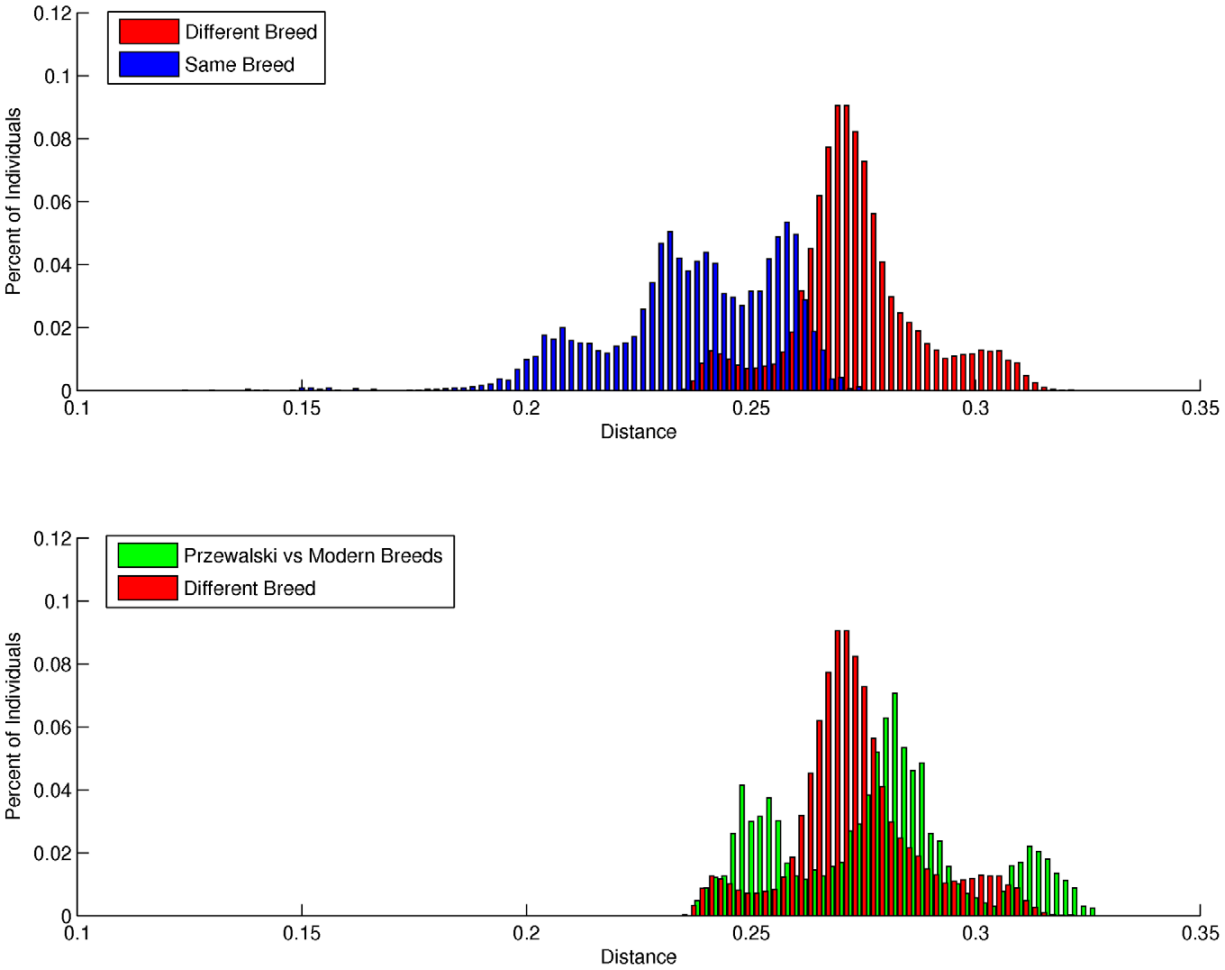
Distribution of pair-wise genetic distances. a), within and between domestic horse breeds; b), between domestic horse breeds and between domestic horse breeds and Przewalski's Horse. Genetic distance (D) between pair-wise combinations of individuals was calculated as described in Materials and Methods.

Mean individual inbreeding coefficients (F) were highest in the Thoroughbred and Standardbred (0.15 and 0.12, respectively), and lowest in the Hanoverian, Quarter Horse and Mongolian (0.06, 0.04, and 0.02, respectively) (Table S9). The average genetic distance (D) between pairs of individuals from different breeds was 0.270 (sd = 0.014), while the mean distance between pairs of individuals from the same breed was 0.240 (sd = 0.020). As seen in Figure 2a, the distribution of D between individuals drawn from different breeds is relatively smooth; however, the distribution of D within breeds is distinctly tri-modal. To further investigate this tri-modal distribution, the mean D was calculated for each breed separately (Table S10). D was lowest in the Norwegian Fjord and Icelandic horses (0.21) which accounted for a large proportion of the left peak in Figure 2a, whereas D was highest in the Hanoverian, Quarter Horse and Swiss Warmblood (0.25–0.26) which accounted for a large proportion of the right peak.

**Figure 3:**
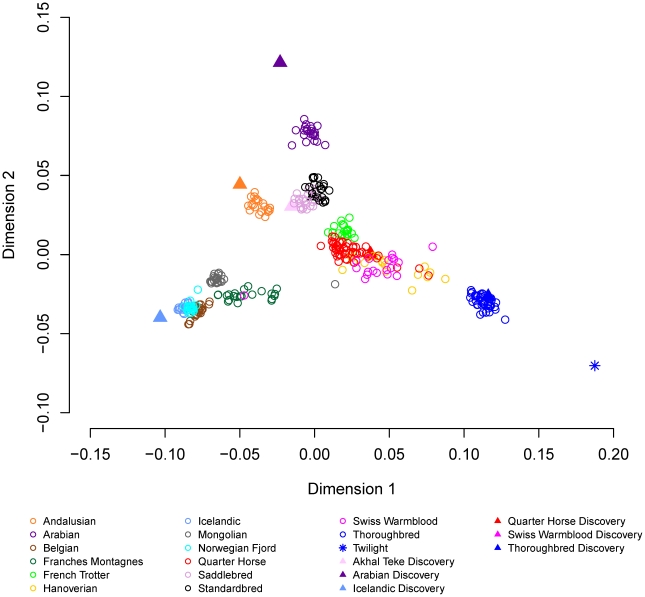
Multidimensional scaling with 14 domestic horse breeds. Metric multidimensional scaling analysis of pair-wise genetic distance was used as described in Materials and Methods to identify relationships between the 14 domestic horse breeds.

Metric multidimensional scaling (MDS) of pair-wise genetic distances was used to visualize the relationships among the 335 horses from 14 breeds. Plotting dimension 1 versus dimension 2 resulted in tight clustering by breed, with the exception of the Quarter Horse, Hanoverian and Swiss Warmblood (Figure 3). The 7 SNP discovery horses and Twilight were outliers relative to other members of their breeds (Figure 3). MDS plots and breed relationships in dimensions 3 through 6 are provided in Figure S3.

### Relationships between the domestic horse and extant Hippomorpha

**Figure 4:**
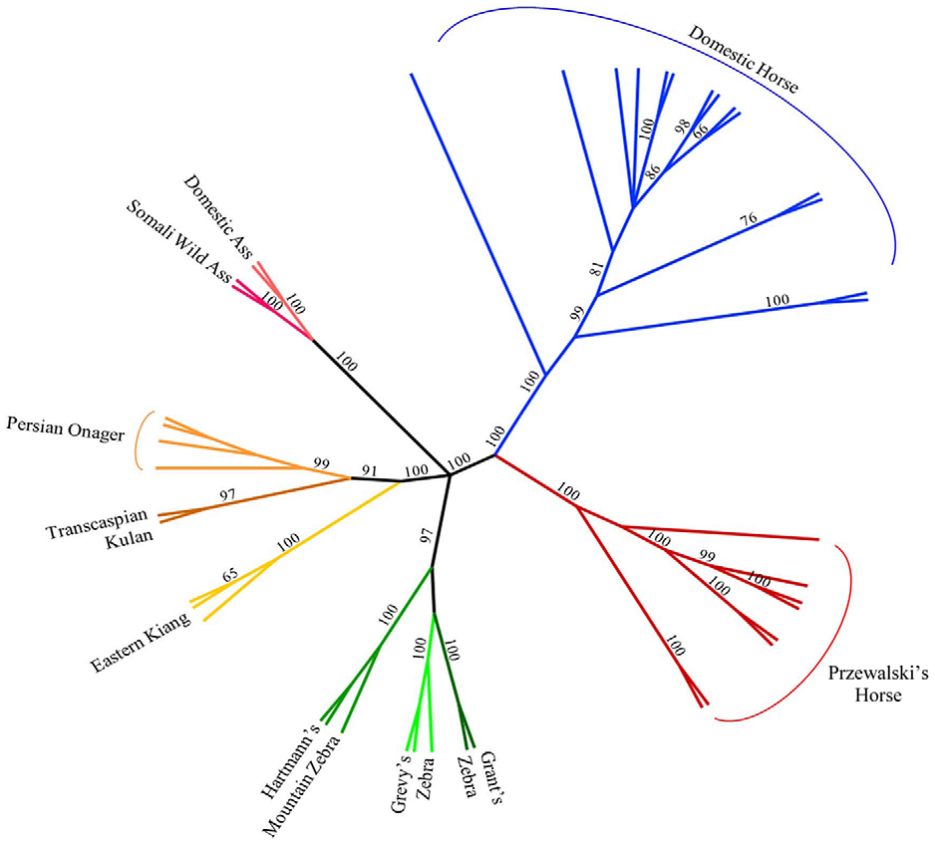
Phylogenetic tree of extant Hippomorpha. Unrooted cladogram constructed via parsimony analysis, considering only the Hippomorpha, using 40,697 autosomal markers. Bootstrap support >50% calculated from 1000 replicates is shown.

Parsimony analysis with a set of 40,697 autosomal SNPs across all Hippomorpha (horses, asses and zebras) placed the modern horse as sister taxa to *Equus przewalski*, and both the modern horse and *E. przewalski* as a sister clade to all the other equids, which fell out into species groups (Figure 4).

Pair-wise genetic distances were also calculated with all domestic horse breeds, and the Przewalski's Horses (n = 9) (Figure 2). MDS revealed tight clustering of the Przewalski's Horse (n = 9) with the Mongolian and Norwegian Fjord horse samples when dimension 1 was plotted against dimensions 2, 3 or 4 (Figures S4 and S5). The Przewalski's Horse samples were not completely separated from the Mongolian and Fjord samples until dimension 6 (Figure S5). The average genetic distance (D) between Przewalski's Horses and domestic horses was greater than the average D between pairs of individuals drawn from any 2 different domestic horse breeds (Figure 2b), however there was significant overlap in the distribution of D values in the Przewalski's-domestic horse pairs and the domestic horse-different breed pairs. To investigate this overlap, the distances between Przewalski's Horses and each breed were calculated (Table S11). The results show that D between Przewalski's Horse and other breeds ranged from 0.25–0.31, and was smaller between Przewalski's Horse and Mongolians, Norwegian Fjords, Belgians and Icelandics than between Przewalski's Horse and Thoroughbreds.

**Figure 5:**
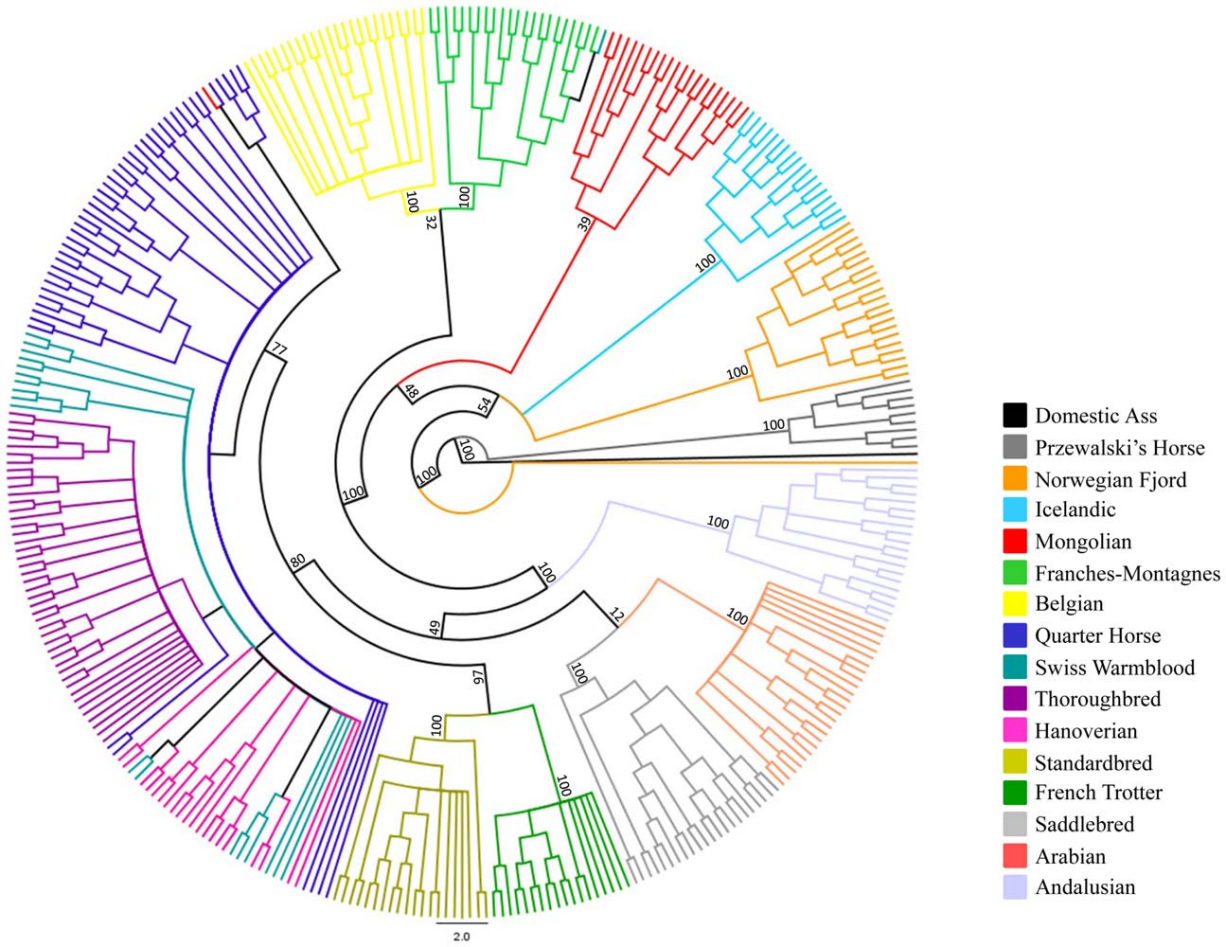
Phylogenetic tree of domestic horse breeds and Przewalski's Horse. Parsimony analysis across 46,244 autosomal loci in the Domestic and Przewalski's Horse. The tree is rooted with the domestic ass.

The relationships between the domestic horse breeds and the Przewalski's Horse are also demonstrated by parsimony analysis in Figure 5, where the Przewalski's Horse falls out into a strongly supported, monophyletic clade that is basal to the remainder of the modern breeds. Parsimony analysis also supports most associations among the domestic horse breeds suggested by MDS (Figure 3).

### Application to genome-wide association studies (GWAS)

**Figure 6:**
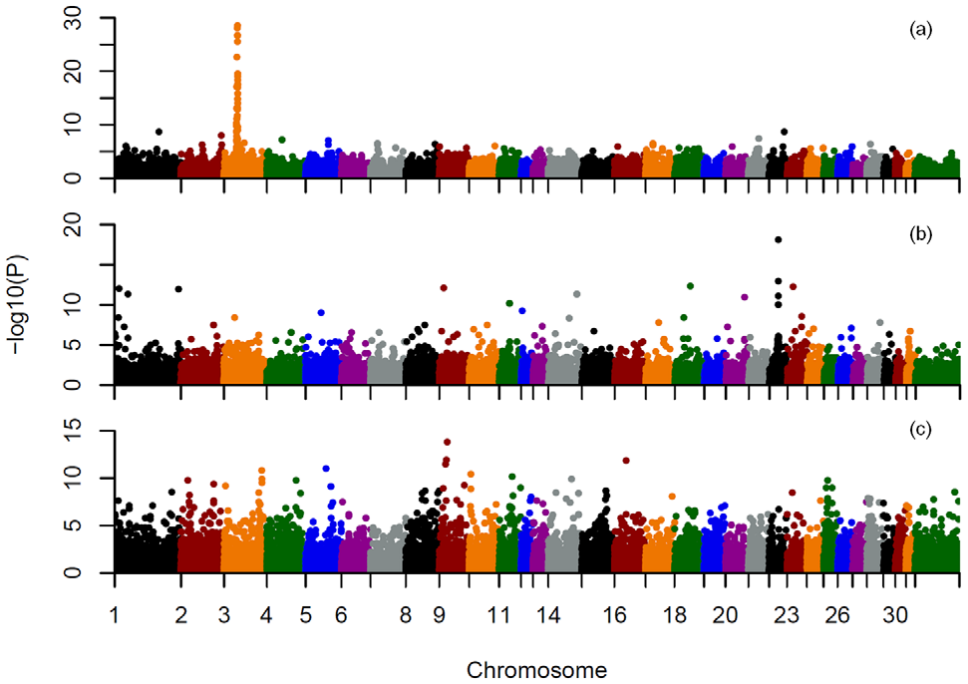
Allele association analysis for three known equine coat color loci. a), mapping of the chestnut locus across breeds, based on inferred coat color phenotype; b), mapping of the black (agouti) locus across breeds, based on inferred black coat color; c), mapping of the gray locus across breeds. Phenotypes were inferred from the genotypes at 9 known coat color loci and known inheritance models as described in Materials and Methods. Unstructured case control association analyses using chi-square tests for allelic association were then performed on a pruned SNP set also as described in Materials and Methods. SNPs on each chromosome are labeled with a different color on the X axis as indicated.

To demonstrate the utility for GWAS, within and across breed mapping was performed for 3 coat color loci. Phenotypes were inferred either from the genotypes of all 9 known coat color loci with consideration of known interactions, or from genotype only at the locus of interest to model a simple Mendelian trait. The three most common alleles in our data set included the recessive chestnut coat color locus (*MC1R*) on ECA3 [5], the recessive black coat color locus (*ASIP*) (agouti) [6] on ECA22, and the dominant gray locus (*STX17*) on ECA25 [7]. Both basic chi-square case-control allelic association and Cochran-Mantel-Haenszel (CMH) association analyses identified the chestnut and black loci across breeds regardless of how phenotype was inferred (Figure 6a and 6b, Tables S12 and S13, and Figures S6 and S7). Gray was not mapped across all breeds using allelic association, CMH, (Figure 6c, Tables S12 and S13, Figure S8), or structured association mapping using principal components or mixed-model analyses to control for underlying population structure (data not shown).

Within breeds the chestnut locus was successfully mapped in Quarter Horses (22 cases and 24 controls) and Thoroughbreds (11 cases and 26 controls) (Table S14). The *ASIP* locus was successfully mapped in the Andalusian (6 cases and 10 controls), when black was considered as a simple recessive trait (ignoring epistatic interactions). The gray phenotype was not successfully mapped within the two breeds attempted.

## Discussion

### Properties of the EquineSNP50 BeadChip

The assay conversion rate was lower on this equine array when compared to a similar assay designed for cattle or pigs (91.0% versus 92.6% and 97.5% respectively); however, SNP validation rates were slightly higher in the horse than in bovine or porcine (98.0% versus 95.1% and 94%, respectively) [8], [9]. SNPs discovered in any two breeds were somewhat more likely to be validated than SNPs discovered in a single breed. Regardless of the discovery source, a large proportion of SNPs were validated and informative in breeds not represented in the SNP discovery effort. In the array design, SNPs from the “across breed” SNP discovery resource rather than SNPs discovered in the genome assembly process were used. This strategy was predicted to increase the utility of the SNP resource in non-Thoroughbred breeds. The high success rate of the validation justifies the approach of using representatives of global breed groups to generate a SNP resource [3]. The 54,000 polymorphic SNPs are distributed across the autosomes with few large gaps (>500 kb). One large gap on ECA6 was the result of a misplaced contig in a pre-release of the sequence assembly from which the array was designed; even though it was correctly placed in the released assembly, no SNPs were selected from this region. Coverage was slightly lower on ECAX, likely reflecting fewer SNPs to choose from for assay design. The SNP discovery algorithm rejects sequences that align equally to multiple locations. The repetitive nature of the X chromosome in most mammals means that this limitation rejects a large number of potential SNPs that would not be positionally informative.

The number and mean MAF of polymorphic SNPs varied between breeds. On average the number of SNPs informative in any given domestic horse breed was higher than informativeness of similar assays within given cattle and dog breeds [8], [10]. Mean MAF across all samples (0.24) was slightly lower than the mean MAF reported for bovine (0.26), ovine (0.28) or porcine assays (0.27) [8], [9] (Illumina Data Sheets at http://www.illumina.com/applications/agriculture/livestock.ilmn#livestock_overview). Breeds with recent or ongoing admixture, such as the Quarter Horse, Hanoverian and Swiss Warmblood, had the highest mean MAF and the largest numbers of informative SNPs, while the lowest mean MAF were in the Norwegian Fjord, Belgian and Icelandic horse. The relatively high number of informative SNPs in the Icelandic horse may reflect its use in SNP discovery. Despite low genetic diversity and high levels of inbreeding, the mean MAF in the Thoroughbred was higher than any other non-admixed breed, and the fraction of informative SNPs exceeded that of any other breed included in the SNP discovery effort with the exception of the Quarter Horse. The high level of SNP informativeness in the Thoroughbred breed likely reflects bias due to its use in both SNP discovery and as the reference genome sequence.

We attempted to use the EquineSNP50 BeadChip to genotype a limited number of individuals from 18 other Perissodactyl species. Due to the SNP discovery design, it was unlikely that a large proportion of the markers in this assay would be polymorphic in other species; however, the identification of even several hundred useful markers in any of these species would provide a dramatic increase in the number of autosomal markers available for conservation genetics applications. A variable number of genotypes were produced across species, with higher genotyping rates and better quality scores in the more closely related Hippomorpha (horses, asses and zebras) than in the Ceratomorpha (rhinos and tapirs). After data filtering, the assay conversion rate of the remaining SNPs was fairly high, and quality scores in Hippomorpha species were similar to those in the domestic horse, suggesting it may be a useful tool for certain applications in species other than *Equus caballus*. SNP validation rates in *Equus* species other than *Equus przewalski* were low, which may reflect species divergence as well as the very limited number of individuals genotyped in most species; genotyping a larger cohort within each species would be necessary to determine the true polymorphism rates. Further work is also necessary to determine the accuracy of genotyping calls in *Equus* sp. by reproducibility, concordance with other genotyping methods and confirmation of Mendelian inheritance with parent-offspring trio data [11]. Lastly, low quality scores, even after data filtering and inconsistent genotyping rates in the Ceratomorpha, suggest that the EquineSNP50 BeadChip will likely have much more limited utility in these species.

### Genetic diversity, inbreeding, and LD in the domestic horse

Measurements of genetic diversity, inbreeding and LD all reflect population demographic history. Our measurements of genome-wide LD within and across breeds agreed well with previous work based on ten randomly selected 2 Mb genome segments [3]. Due to population subdivision, the extent of LD within a given breed was greater than LD across breeds. LD in the domestic horse is lower than in dogs, which does not decline nearly as rapidly over the first 100 kb and has a very slow decline over the next 1–2 Mb [12]. Not surprisingly, within breed patterns of LD in horses were similar to those observed in domestic cattle, which typically share a similar system of mating using popular sires and at times extensive line breeding [13].

LD declined most rapidly in the Quarter Horse and Mongolian horse, with *r^2^* values dropping below 0.2 within the first 50–100 kb. The short extent of LD in the Mongolian and Quarter Horse reflects the low level of inbreeding and high genetic diversity in both breeds. The short extent of LD in the Mongolian horse is likely a result of its age and large population size. This breed has been bred in domestication since approximately 2000 BC, and the current population size is ∼3 million individuals [2]. High diversity in the Mongolian horse is in concordance with previous studies based on microsatellite loci that demonstrated that the Mongolian horse had the highest heterozygosity and genetic diversity in a study of 13 domestic horse populations [14]. Unlike the Mongolian horse, other breeds with long histories had a moderate decline in LD. These include the Icelandic horse, which originated from stock imported to Iceland in ∼900 AD, the Norwegian Fjord horse thought to have been selectively bred for at least 2,000 years, and the Belgian draft horse believed to be descended from the war horse of the Middle ages [2]. Somewhat longer LD in these old breeds likely reflects the fact that their population histories have included severe population bottlenecks. An Icelandic horse bottleneck has been associated with the 1783 eruption of the volcano Lakagigar, in which an estimated 70% of the population was destroyed from volcanic ash poisoning [2], and a population bottleneck in the Belgian and other draft horse breeds arose due to their disappearance as a utilitarian animal after World War II. It has been postulated that all present day Norwegian Fjords are descendants of a single stallion foaled in 1891, however previous studies using microsatellite markers have not yet corroborated this assumed bottleneck [15]. Short LD in the Quarter Horse, a recently established breed with a registry less than 100 years old, is likely a result of a very large population size (∼4 million individuals), rapid population expansion and population admixture since the breed's formation [16], [17].

In contrast, LD was clearly the highest in the Thoroughbred, reflecting the breed's low diversity, high inbreeding, and closure of the studbook to outside genetic influence for more than 300 years. Previous work has demonstrated that approximately 78% of Thoroughbred alleles are derived from 30 founders, and that a single founder stallion is responsible for approximately 95% of paternal lineages [18]. The long extent of LD in this breed also reflects the high level of inbreeding which has been shown to have an even greater impact on the extent of LD than diversity [17]. The impact of low diversity and high inbreeding on LD can also be seen in the Standardbred and the French Trotter, both breeds which, while having a more rapid decline in LD than the Thoroughbred, have long-range LD that persists further than the Thoroughbred.

### Relationships between domestic horse breeds

The mean pair-wise genetic distance between individuals within a breed was 0.24, which is higher than reported in cattle, but lower than reported in sheep (0.21 and 0.25 respectively) [19]. However, D was not normally distributed in horses, displaying three distinct peaks. When the distance matrix was partitioned by breed, the pair-wise distances were largest within the Quarter Horse, Swiss Warmblood and Hanoverian, all breeds with admixture and low to moderate levels of inbreeding, while the pair-wise distances were the smallest within the Norwegian Fjord and Icelandic horse, which may reflect their previous population bottlenecks. There is also substantial overlap between the within and across breed distributions, which was likely the result of high genetic diversity in admixed breeds, as well as close relationships between breeds such as the Standardbred and French Trotters.

MDS plots demonstrated that individuals within most breeds were tightly clustered in relation to other breed groups. This was true even for the Thoroughbred population where two geographically distinct sample origins were represented (United Kingdom, Ireland, and United States). The exceptions to this were the three breeds with recent and/or ongoing admixture; the Quarter Horse, Hanoverian and Swiss Warmblood. In addition, the Hanoverian and Quarter Horse, and to a lesser extent the Swiss Warmblood, had larger variation along dimension 1 than other breeds, suggesting that the admixture may be resulting in significant population substructure. The Andalusian breed was not tightly clustered in dimension 6, suggesting population substructure as well. This is consistent with the practice of some American breeders crossing Andalusians (from Spain) with closely related Lusitano horses (from Portugal) in their breeding programs. Close relationships between some breeds were also visualized, including the clustering of the Standardbred and the French Trotter apart from the other breeds in dimension 3. This may be the result of the influence of the Standardbred on the French Trotter, or similar selective pressures for the trotting phenotype in both breeds. The Norwegian Fjord, Icelandic, Mongolian, and Belgian clustered together in the first 3 dimensions, and Icelandic and Norwegian Fjord horses clustered tightly together in all 6 dimensions. This may reflect the suggested influence of Mongolian genes in the development of the Norwegian Fjord and subsequent development of the Icelandic horse from Scandinavian stock imported to Iceland [15], [20]. However the close clustering of the Belgian horse with these older breeds does not fit this history and its clustering may also reflect the low MAF and lower number of informative SNPs in the Belgian, Icelandic and Norwegian Fjord. Ten horses are outliers relative to their breed: a Norwegian Fjord, a Mongolian, the seven SNP discovery horses, and Twilight. Increased heterozygosity due to SNP discovery bias likely accounts for the outlier status of Twilight and the seven SNP discovery horses. We expect to observe greater diversity in all SNP discovery breeds because observations of diversity in other breeds rely on across-breed allele sharing rather than direct allelic observation. Parsimony analysis supports many relationships suggested by MDS. For instance, breeds in which individuals cluster tightly in MDS, such as the Thoroughbred and Arabian, are represented in the cladogram as monophyletic clades with high bootstrap support; whereas breeds that have continuing admixture, such as the Quarter Horse, Swiss Warmblood, and Hanoverian, do not show monophyly and share a branch of the clade with the Thoroughbred. In some instances, relationships that were not clear from the MDS plot are demonstrated in the tree, such as the close placement of the Saddlebred and Arabian.

### Relationship between the domestic horse and other Hippomorpha

In parsimony analysis of only *Equus* spp. using over 40,000 SNPs, high bootstrap support distinguishes *Equus caballus* from *Equus przewalskii* while also making a clear distinction between those species and the zebras and asses. With further work, the use of random nuclear SNPs in equid phylogeny studies should prove superior to the existing studies that use either mitochondrial SNPs, or SNPs from just a few nuclear genes [21]–[23].

The horse is thought to have been domesticated from the now extinct Tarpan (also known as the European wild horse *Equus ferus*) [1]. The close clustering of the domestic horse and the Przewalski's Horse is consistent with the hypothesis that the Przewalski's Horse (also known as the Asiatic wild horse *Equus przewalskii*) is a sister species to the Tarpan. This close relationship between the domestic horse and the Przewalski's Horse is also likely a result of relatively recent gene flow between these lineages since divergence from a common ancestor. While *Equus przewalskii* and *Equus caballus* have a different number of chromosomes (2n = 66 and 2n = 64, respectively), they can interbreed and produce viable offspring. Since their discovery by the western world in the late 1880s, the question of admixture of the Przewalski's Horse and domestic horse has remained a topic of debate and controversy. Known introgressions took place in the early years of the propagation program that prevented the extinction of the species [24] and, more recently with the offspring of the last wild-caught mare at the Askania Nova breeding center [25]. In addition, there was likely interbreeding of *Equus przewalskii* and *Equus caballus* in the wild, as the range of the Przewalski's Horse and the domestic horse overlapped in China, Russia and Mongolia [26]. Gene flow from the domestic to Przewalski's Horse in our study is supported by the tight clustering of the Przewalski's Horse and several of the horse breeds in MDS, most notably the Mongolian horse and related breeds. This relationship is reiterated by parsimony analysis where the Mongolian, Icelandic, and Norwegian Fjord are in close association with the Przewalski's Horse. The pair-wise genetic distances between Przewalski's Horses and some domestic horse breeds falls within the range of within breed pair-wise differences in domestic horse breeds, which corroborates earlier findings [3]. Thus, while *Equus ferus* and *Equus przewalskii* are considered different species based on chromosomal number differences, surviving Przewalski's Horses today are truly *Equus przewalskii* and *Equus caballus* hybrids [1].

### Application to genome-wide association studies

A major application of this genotyping technology will be in genome-wide association mapping of traits in the domestic horse [27]–[31]. The success of such studies will depend upon LD within the mapping population, properties of the loci themselves, population structure, and the mode of inheritance. Our attempt to map three known Mendelian coat color traits in a sample set not specifically designed for that purpose, met with varying success (Tables S12, S13, S14; Figures S6, S7, S8).

The *MC1R* locus was successfully mapped both across breeds and within several breeds. This is a result of good informative SNP density in this region, larger sample sizes for several breeds in which the chestnut allele is segregating, and extended homozygosity surrounding the locus. The centromeric location of the *MC1R* locus that limits recombination, as well as selection for the chestnut trait in many breeds, resulted in a conserved haplotype within breeds ranging from 1.2–4.2 Mb and a 750 kb minimally conserved haplotype across breeds (Figure S9, Table S15). The length of this conserved haplotype is nevertheless surprising given the presence of the *MC1R* chestnut allele since at least the fifth millennium before present [32] On the other hand, the mapping of *ASIP*, while successful across breeds, suffered from lower numbers of relevant samples within many individual breeds and low SNP density at the *ASIP* locus itself. Mapping the *STX17* gray locus was unsuccessful due to confounding by population substructure, sparse marker density in the region, and poor power to detect a dominant locus due to low sample sizes both within and across breeds. Nevertheless, our results demonstrate the utility of whole genome mapping within breeds when studies are sufficiently powered, although power clearly varies among breeds, and the rate of false positives increases with small sample sizes. Further, due to across-breed haplotype sharing in the horse [3], across-breed mapping of certain traits that are clearly conserved across breeds is possible if proper consideration is given to confounding population substructure. Ideally, increased genome coverage with additional, highly informative SNPs would be more effective for mapping studies, particularly in admixed and/or breeds with low LD.

### Concluding statement

We have constructed and validated a 54,000 SNP genotyping assay that will enable mapping of loci associated with equine health and performance, as well as the study of breed diversity and relationships. The array will also likely have many uses in the study of the population genetics of other equid species.

## Materials and Methods

### Design of the EquineSNP50 BeadChip

SNPs assayed on the EquineSNP50 BeadChip were selected from the “across breed” SNP set generated in the equine genome project. The SNPs used for the genotyping array had unknown validation status and minor allele frequency at the time of design. The discovery method used in the equine genome analysis (SSAHA-SNP) predicted a 98% validation rate. SNP quality scoring was conducted by Illumina Inc. and included estimates of sequencing quality on the Illumina platform based on flanking characteristics such as G-C content, proximity of known SNPs and unique flanking sequence. After scoring, SNPs requiring a single-bead type (Infinium II) were preferred and of these, the highest scoring 60,000 were passed to the array design regardless of genomic location.

### Horses

Twilight and the 7 SNP discovery horses from the NHGRI equine genome sequencing project (an Andalusian, Arabian, Akhal-Teke, Icelandic, Standardbred, Thoroughbred and a Quarter Horse) were selected as controls [3], and each of these horses was genotyped in duplicate. The remaining 346 genotyped horses were from 14 different breeds (Table S2). Breeds were selected where possible to represent a geographic distribution from Europe, Asia and North America. 18 mare-stallion-foal trios were genotyped from 12 different breeds (Table S3). Three of the 6 Thoroughbred trios had the same sire, while two Thoroughbred trios, and two Belgian trios shared the same sire and dam (*i.e.* full-sibling pairs). The remaining individuals from any given breed were selected to be no more related than second cousins (*i.e.*, not sharing grandparents) based on pedigree analysis.

### Samples from other Perissodactyla

The utility of the EquineSNP50 BeadChip for use in other Perissodactyla was determined by assaying the 54,602 SNPs that produced genotypes in *Equus caballus* in 53 individuals from 18 species evolutionarily related to the domestic horse, including domestic and wild asses, zebras, tapirs and rhinoceroses (Table S7).

### SNP summary statistics

Individual genotyping rate was defined as the proportion of loci that produced a genotype in that individual. SNP conversion rate was calculated as the number of SNPs that produced a genotype/number of SNPs included in the assay. Validation rate was calculated as the number of polymorphic SNPs (at least 1 heterozygous individual)/number of converted SNPs. Mendelian errors in each of the 18 nuclear trios were calculated using PLINK [33] and results are reported as Mendelian agreement (1-[number of Mendelian errors in the trios/total number of loci genotyped]). Informative SNPs were defined as those SNPs with a minor allele frequency greater than 0.05. Minor allele frequencies and missingness rates were calculated using PLINK (–freq, –missing) [33]. The proportion of validated SNPs from each discovery breed was computed by identifying all SNPs within the assay that were discovered in a single breed (relative to Twilight's sequence) and determining the proportion of those SNPs that were polymorphic within each of the 14 breeds included in the sample set (with the exclusion of the discovery horses). Genotyping quality for each call was determined using the GenCall method in Illumina's Genome Studio software [11]. GenCall (GC) scores are reported as the 10^th^ percentile of the GC scores (GC10 scores).

### Additional data filtering in extant Perissodactyla

Additional filtering criteria were applied to the genotyping data in extant Perissodactyla, First, calls with low signal intensity were identified by combining the X raw and Y raw intensity values for both allele A and allele B, and removing genotypes failing to reach the threshold value (set at 1000). These loci were then removed and re-coded as no calls (00) in the ped file for further analyses. After intensity filtering, individual genotyping rates were determined. Mean and standard deviations for individual genotyping rates were determined for both the Hippomorpha and the Ceratomorpha. Individuals with genotyping rates below 2 standard deviations from the mean were excluded from further analyses. Markers that genotyped in >90% of the remaining individuals were considered true markers and are reported as loci producing a genotype in the species (Table S8).

### Genome-wide linkage disequilibrium

Genome-wide LD was estimated by calculating *r^2^* values between all pairs of SNPs with inter-SNP distances of less than 4 Mb both within a given breed (a minimum of 18 horses per breed) and across all breeds. Pair-wise LD was calculated for each chromosome within breed using the LD plot function in Haploview [34] exporting the data to text files. These files were computationally processed to produce mean *r^2^* values in 50 Kb distance bins across all chromosomes for individual breeds and for all horses. Inter-SNP distances of greater than 4 Mb were ignored. Extent of LD was regarded as the persistence of LD until falling below two-fold background LD. Background LD within breed is largely affected by sample size and effective population size.

### Relationship between breeds and genetic structure in the domestic horse

Genetic distance (D) between pair-wise combinations of individuals was calculated using PLINK where D = 1−[(IBS2+0.5*IBS1)/N]: IBS2 and IBS1 are the number of loci that share either 2 or 1 alleles identical by state (IBS), respectively, and N is the number of loci tested. Metric multidimensional scaling (MDS) analysis of pair-wise genetic distance (6 dimensions) was used to identify the relationships between breeds with PLINK (–mds-plot 6).

### Estimation of inbreeding

Individual inbreeding coefficients (F) were estimated with PLINK. SNPs were pruned for linkage equilibrium using pair-wise genotypic correlation in 100 SNP windows sliding by 25 SNPs along the genome; SNPs were pruned at *r^2^*>0.2 (–indep-pairwise 100 25 0.2). The resulting set of 17,947 SNP loci was used to calculate F for all individuals within each breed.

### Relationship among the domestic horse breeds and the Prezwalski's horse

Genetic distance (D) between pair-wise combinations of individuals was calculated and MDS analysis of pair-wise genetic distance (6 dimensions) was used to identify the relationships using PLINK (as above). D and MDS calculations for the domestic horse and Przewalski's Horse group included all domestic horses as well as the control horses.

Autosomal genotypes from 344 individuals of the 14 modern breeds, the 9 Przewalski's Horses, and a domestic ass, were pruned in PLINK for a minimum per-SNP genotyping rate of 0.9 (–geno 0.1) and minor allele frequency of 0.05 (–maf 0.05). Alleles of the 46,244 remaining SNPs were coded AA = 0, AB = 1, and BB = 2 for parsimony analyses in TNT (Goloboff et al. 2003) with a domestic ass designated as the outgroup. Traditional searches were applied using subtree pruning-regrafting (SPR) branch swapping for 100 replicates followed by 100 replicates of the tree bisection-reconnection (TBR) method. New technology searches were them performed (random and consensus sectoral searches, 5 rounds of tree fusing, and 30 iterations of tree-drifting), at default settings. Bootstrap support was calculated using 500 pseudoreplicates with traditional search methods (SPR-TBR). The resulting tree was visualized in FigTree (http://tree.bio.ed.ac.uk/software/figtree/).

### Relationship among the extant equids and the domestic horse

Phylogeny across the Hippomorpha was performed in TNT similarly to the phylogenetic analysis above [35]. The X chromosome was removed and autosomal SNPs were pruned for a per-locus genotyping rate of ≥90% (–geno 0.1) and maf >0.0001 across species, and an unrooted cladogram was created from parsimony analysis of the remaining in 40,697 autosomal markers. Bootstrap support was calculated from 1000 replicates.

### Mapping of coat color loci

All horses were genotyped for all known coat color loci including *MC1R* (chestnut), *ASIP* (agouti, black/bay), *STX17* (gray), *SLC36A1* (champagne), *MATP* (cream), *PMEL17* (silver), *KIT* [exon skipping] (sabino), *EDNRB* (overo) and *KIT* [inversion] (tobanio) [5]–[7], [36]–[42]. Genotypes were determined using methods routinely employed at the Veterinary Genetics Laboratory, University of California Davis. The base coat color phenotype of each horse was determined either by inference from the genotypes of all 9 coat color loci with consideration of known interactions, or by inference from genotype only at the locus of interest (*MC1R*, *ASIP*, or *STX17*) to model a simple Mendelian trait. To infer coat color phenotype based on the 9 known loci:

1. The base color of the horse was determined by *MC1R* genotype. Horses homozygous for the mutant *MC1R* allele (M/M) were classified as chestnut. Those with one or more copies of the wild type allele (E/M, E/E) were considered black-pigmented.
2. Considering only non-chestnut horses, those homozygous for the exon 2 deletion in *ASIP* were classified as black, while those with one or two copies of the wild type allele were considered bay.
3. All other modifying loci were then considered which would result in dilution (silver, champagne, cream) or white patterning (sabino, overo, tobiano), and horses were assigned appropriate coat color phenotypes (9 palomino, 8 buckskin, 1 cremello, 1 smoky black/champagne, and 4 with white patterning).
4. All horses with the *STX17* exon 6 duplication were classified as gray, due to the epistatic dominance of the gray coat color over all other loci.
5. Norwegian Fjord horses were all considered to have the dun coat color due to fixation of this coat color in the breed and the lack of a genetic test for dun.

Coat color association analyses were performed in PLINK [33], and quality control summary statistics including genotyping rate, MAF, Hardy-Weinberg equilibrium tests and case/control differences in genotype missingness were performed for all analyses. SNPs with MAF<1.0%, genotyping rate <90%, and individuals genotyped at a rate of <90% were excluded from further analysis. Additionally, SNPs were excluded if they demonstrated deviation from HWE (p<0.001), or differential missingness between cases and controls (p<0.01). Multiple testing correction, when performed, was accomplished with 10,000 label swapping t-max permutations (–mperm 10,000). T-max permuted p-values were considered genome-wide significant at p<0.05. Inflation of p-values due to population structure was assessed by calculating the genomic inflation factor (λ) and by assessing quantile-quantile plots.

Unstructured genome-wide association analysis was performed using chi-square tests for allelic association (–assoc). Stratified genome-wide association analysis was performed using the Cochran-Mantel-Haenszel (CMH) test (–mh). Horses were clustered for the CMH test on the basis of the pair-wise population concordance (PPC) test in PLINK, which clusters individuals based on the likelihood of concordant or discordant ancestry. A p-value for merging in the PPC test was set at p = 0.01 (–cluster, –ppc 0.01). Manhattan plots of all results were generated using Haploview [34].

### Haplotypes at coat color loci

Haplotypes containing markers at the *MC1R*, *ASIP* and *STX17* loci on ECA3, 22 and 25, respectively, were determined with fastPHASE [43]. For *MC1R*, the number of chromosomes in each breed that contained the chestnut allele, the number of SNPs on which the haplotype is based, and the length and coordinates of the shared haplotype are available in Table S15. Similar analyses resulted in no shared haplotypes at either the *ASIP* or *STX17* loci.

## Supporting Information

Figure S1 Distance between informative SNPs across all 31 autosomes. Informative SNPs were defined as having MAF>0.05 across all 14 breeds.
(TIF)

Figure S2 Decline in genome-wide linkage disequilibrium across and within breeds. Genome-wide linkage disequilibrium (LD) was estimated both within a given breed, and across all breeds, by calculating *r^2^* values between all pairs of SNPs with inter-SNP distances of less than 600 kb as described in Materials and Methods.
(TIF)

Figure S3 Multidimensional scaling with 14 domestic horse breeds. Metric multidimensional scaling analysis of pair-wise genetic distance was used as described in Materials and Methods to identify relationships between the 14 domestic horse breeds. In these plots dimensions 3–6 (y axes) are always plotted against dimension 1 (x axis).
(TIF)

Figure S4 Multidimensional scaling of Przewalski's Horse and domestic horse breeds. Metric multidimensional scaling analysis of pair-wise genetic distance was used as described in Materials and Methods to identify relationships between the 14 domestic breeds and the Przewalski's Horse.
(TIF)

Figure S5 Multidimensional scaling of Przewalski's Horse and domestic horse breeds. Metric multidimensional scaling analysis of pair-wise genetic distance was used as described in Materials and Methods to identify relationships between the 14 domestic breeds and the Przewalski's Horse. In these plots dimensions 3–6 (y axes) are plotted against dimension 1 (x axis).
(TIF)

Figure S6 Mapping of the chestnut coat color locus across breeds based on phenotype inferred from all nine genotyped coat color alleles with known interactions (a), or based solely on *MC1R* genotype and chestnut as a simple recessive trait (b). Phenotypes were inferred as described in Materials and Methods. Unstructured case-control association analyses using chi-square tests for allelic association were then performed on a pruned SNP set also as described in Materials and Methods. SNPs on each chromosome are labeled with a different color on the X axis as indicated. Also indicated on the X axis is the number of pruned SNPs across the genome included in the analysis.
(TIF)

Figure S7 Mapping of the recessive black coat color locus across breeds based on phenotype inferred from all nine genotyped coat color alleles with known interactions (a), or based solely on *ASIP* genotype and black color as a simple trait (b). Phenotypes were inferred as described in Materials and Methods. Unstructured case-control association analyses using chi-square tests for allelic association were then performed on a pruned SNP set also as described in Materials and Methods. SNPs on each chromosome are labeled with a different color on the X axis as indicated. Also indicated on the X axis is the number of pruned SNPs across the genome included in the analysis.
(TIF)

Figure S8 Mapping of the gray coat color locus across breeds based on gray color as a simple dominant trait. Gray phenotype was inferred as described in Materials and Methods. Corrected p-values after 10,000 label-swapping permutations are indicated. SNPs on each chromosome are labeled with a different color on the X axis as indicated. Also indicated on the X axis is the number of pruned SNPs across the genome included in the analysis.
(TIF)

Figure S9 Conserved haplotype at the *MC1R* locus on ECA3. For all breeds with the exception of the Standardbred, the length of minimal homozygosity for the chestnut allele is depicted. In the Standardbred, where no homozygotes were observed, the shortest length of the haplotype containing the chestnut allele is depicted. The number of chromosomes in each breed that contained the chestnut allele ranged from 7 in the Andalusian, and 10 in the Standardbred and Norwegian Fjord, to 41 in the Saddlebred and Thoroughbred, 48 in the Belgian and 75 in Quarter Horse, with an average of 27.8 across all 14 breeds. The number of SNPs on which the haplotype is based ranged from 16 in the Andalusian to 75 in the Standardbred, with an average of 37.8 across all 14 breeds. The length of the shared haplotype ranged from 1.08 Mb in Andalusian to 4.157 Mb in French Trotter, with an average length of 2.16 Mb across all 14 breeds. The complete list of chromosome numbers, numbers of SNPs on which the haplotype is based, and the length and coordinates of the shared haplotype are available in Table S13. The position of *MC1R* and the likely position of the centromere are depicted on the figure. Similar analyses resulted in no shared haplotypes for either *ASIP* or grey.
(TIF)

Table S1 Validation rates for the assayed SNPs. The number of selected SNPs from the seven discovery horses and Twilight that provided genotypes (converted), the number of validated SNPs in the equine sample set, and the validation fraction are indicated. SNPs with the highest validation rate were those originally ascertained in two breeds, especially if one of the horses was Twilight. SNPs with the lowest validation rate were originally ascertained in three breeds but were not present in Twilight's sequence.
(DOCX)

Table S2 Breeds included in the study. Breed, number of individuals, and geographic origin of horses genotyped in this study. *The control horses consisted of the 7 SNP discovery horses listed in Table S1 and the Thoroughbred mare Twilight used to generate the equine genome assembly.
(DOCX)

Table S3 Tests of Mendelian inheritance in trios. Mendelian errors in each of the 18 nuclear trios were calculated and results are reported as Mendelian agreement as described in the Materials and Methods.
(DOCX)

Table S4 Inter-SNP spacing by chromosome. Polymorphic SNPs were defined as having at least one heterozygous individual (*i.e.*, MAF>0) across all 14 breeds.
(DOCX)

Table S5 Allele frequencies and genetic diversity in the domestic horse. The mean and median MAF (including all SNPs), the number of polymorphic (MAF≥0.01) and informative SNPs (MAF>0.05), and genetic diversity as determined by heterozygosity (H_E_), are indicated and calculated as described in Materials and Methods.
(DOCX)

Table S6 Proportion of validated SNPs from each discovery breed across all breeds genotyped. The impact of discovery breed on SNP validation rate across the genotyped sample set was determined as described in Materials and Methods.
(DOCX)

Table S7 Species, genotyping rates, sample and locus GC scores for unfiltered data in extant Perissodactyla. Genotyping rates and GC scores reported using Illumina's default calling parameters as described in Materials and Methods.
(DOCX)

Table S8 Quality scores, SNP conversion and SNP validation rates and mean heterozygosity for extant Perissodactyla after filtering for intensity and genotyping rate. Genotyping rates, conversion and validation rates and observed hetrozygosities after filtering the data as described in Materials and Methods. Conversion and validation rates and heterozygosities are not reported for Ceratomorpha due to low genotyping quality.
(DOCX)

Table S9 Inbreeding coefficients in each breed. Individual inbreeding coefficients (F) were estimated with 17,947 SNPs that were pruned for linkage equilibrium as described in Materials and Methods.
(DOCX)

Table S10 Mean pair-wise genetic distances in domestic horse populations. Genetic distance (D) between pair-wise combinations of individuals was calculated as described in Materials and Methods.
(DOCX)

Table S11 Mean pair-wise distances between Przewalski's Horse and domestic horse populations. Genetic distance (D) between pair-wise combinations of individuals was calculated as described in Materials and Methods.
(DOCX)

Table S12 Chi square analysis for mapping of known coat color loci across the 14 breeds. Chestnut, black and gray phenotypes were either inferred from the genotypes at 9 known coat color loci, or from the genotype of a single locus (designated as *MC1R* and *ASIP*) only, using known inheritance models as described in Materials and Methods. Case control association analyses were then performed on a pruned SNP set also as described in Materials and Methods. The genomic inflation factor lambda, the number of SNPs with an EMP2<0.05 after 10000 label-swapping permutations, the number of these SNPs within 5 Mb of the true locus (true positive SNPs), the length of the chromosomal segment at the true gene locus containing true positive SNPs, and the false discovery rate (percentage of all positive SNPs that are within 5 Mb of the true locus), are all indicated.
(DOC)

Table S13 CMH analysis for mapping of known coat color loci across the 14 breeds. Phenotypes were inferred from multi or single locus genotypes as described in Table S1 and the Materials and Methods. Stratified genome-wide association analysis was performed using the Cochran-Mantel-Haenszel (CMH) test. Horses were clustered for this test on the basis of the pair-wise population concordance test also as described in Materials and Methods. The genomic inflation factor lambda, the number of SNPs with an EMP2<0.05 after 10000 label-swapping permutations, the number of these SNPs within 5 Mb of the true locus (true positive SNPs), the length of the chromosomal segment at the true gene locus containing true positive SNPs, and the false discovery rate (percentage of all positive SNPs that are within 5 Mb of the true locus), are all indicated.
(DOC)

Table S14 Chi-square tests of association for coat color loci within breeds. Chestnut, black and gray phenotypes were either inferred from the genotypes at 8 known coat color loci, or from the genotype of a single locus (designated as *MC1R* and *ASIP*) only, using known inheritance models as described in Materials and Methods. Case-control association analyses were then performed on a pruned SNP set also as described in Materials and Methods. The genomic inflation factor lambda, the number of SNPs with an EMP2<0.05 after 10000 label-swapping permutations, the number of these SNPs within 5 Mb of the true locus, the lowest EMP2 value (if <1.0), and the chromosomal location of the SNP with the lowest EMP2 value, are all indicated.
(DOC)

Table S15 Summary of *MC1R* Haplotype Analysis on ECA3. Genotype data was phased as described in Materials and Methods and summary statistics on the chromosomes containing the associated haplotype are presented by breed.
(DOCX)

Text S1 Supporting text includes additional information on: filtering of genotypes in Perissodactyla, the relationships between domestic horse breeds and between the domestic horse and the Przewalski's Horse, and genome wide association analysis.
(DOCX)
