# Supplementary figures and images for: A High Density SNP Array for the Domestic Horse and Extant Perissodactyla: Utility for Association Mapping, Genetic Diversity, and Phylogeny Studies
Source: PLoS Genet. 2012 Jan 12;8(1):e1002451. doi: 10.1371/journal.pgen.1002451 (PMC3257288; doi:10.1371/journal.pgen.1002451)

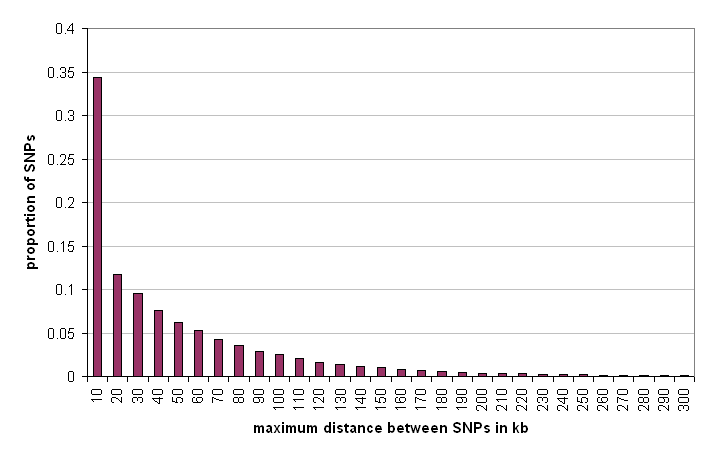

Supplement: Figure S1 — Distance between informative SNPs across all 31 autosomes. Informative SNPs were defined as having MAF>0.05 across all 14 breeds. (TIF) [file pgen.1002451.s001.tif]

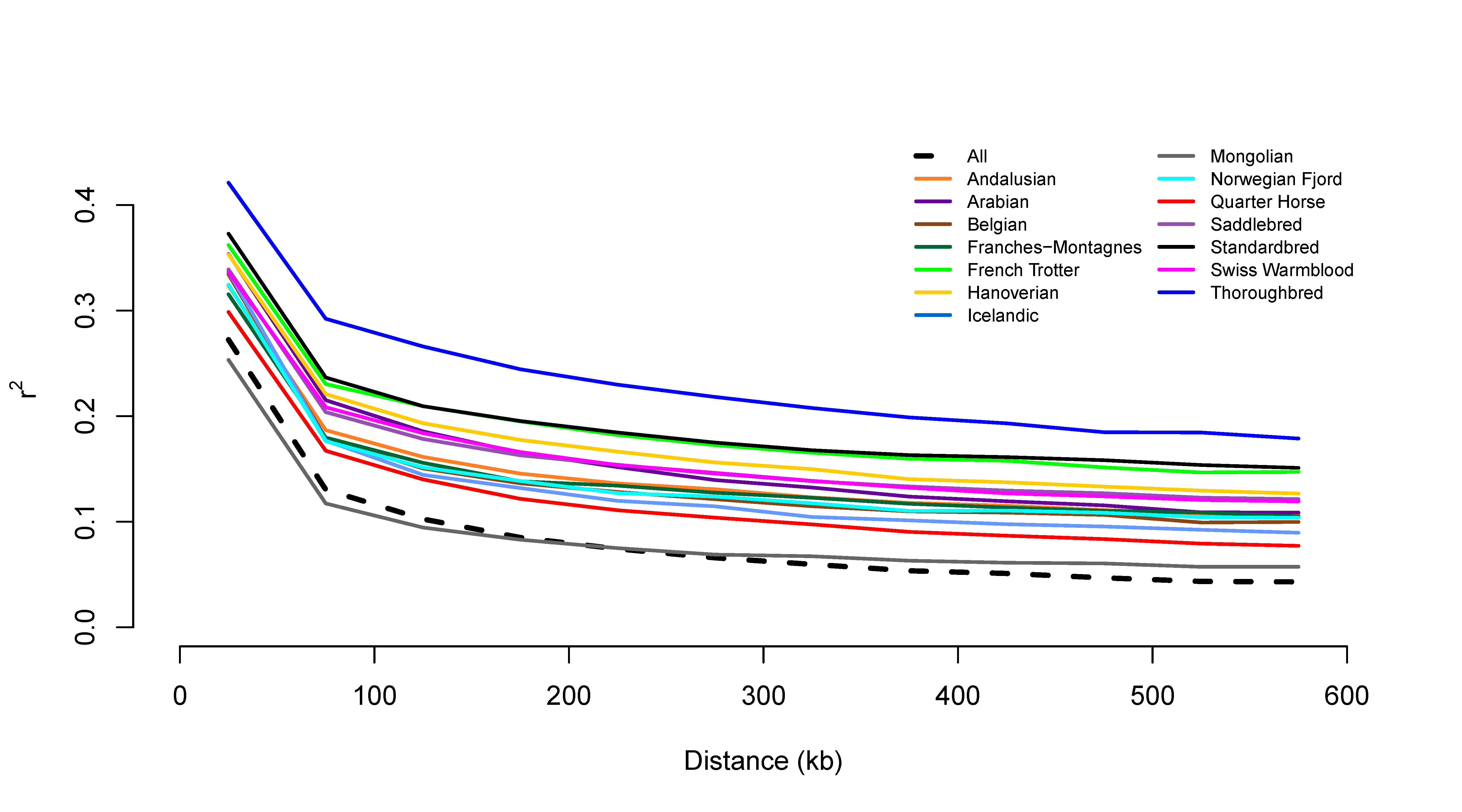

Supplement: Figure S2 — Decline in genome-wide linkage disequilibrium across and within breeds. Genome-wide linkage disequilibrium (LD) was estimated both within a given breed, and across all breeds, by calculating r2 values between all pairs of SNPs with inter-SNP distances of less than 600 kb as described in Materials and Methods. (TIF) [file pgen.1002451.s002.tif]

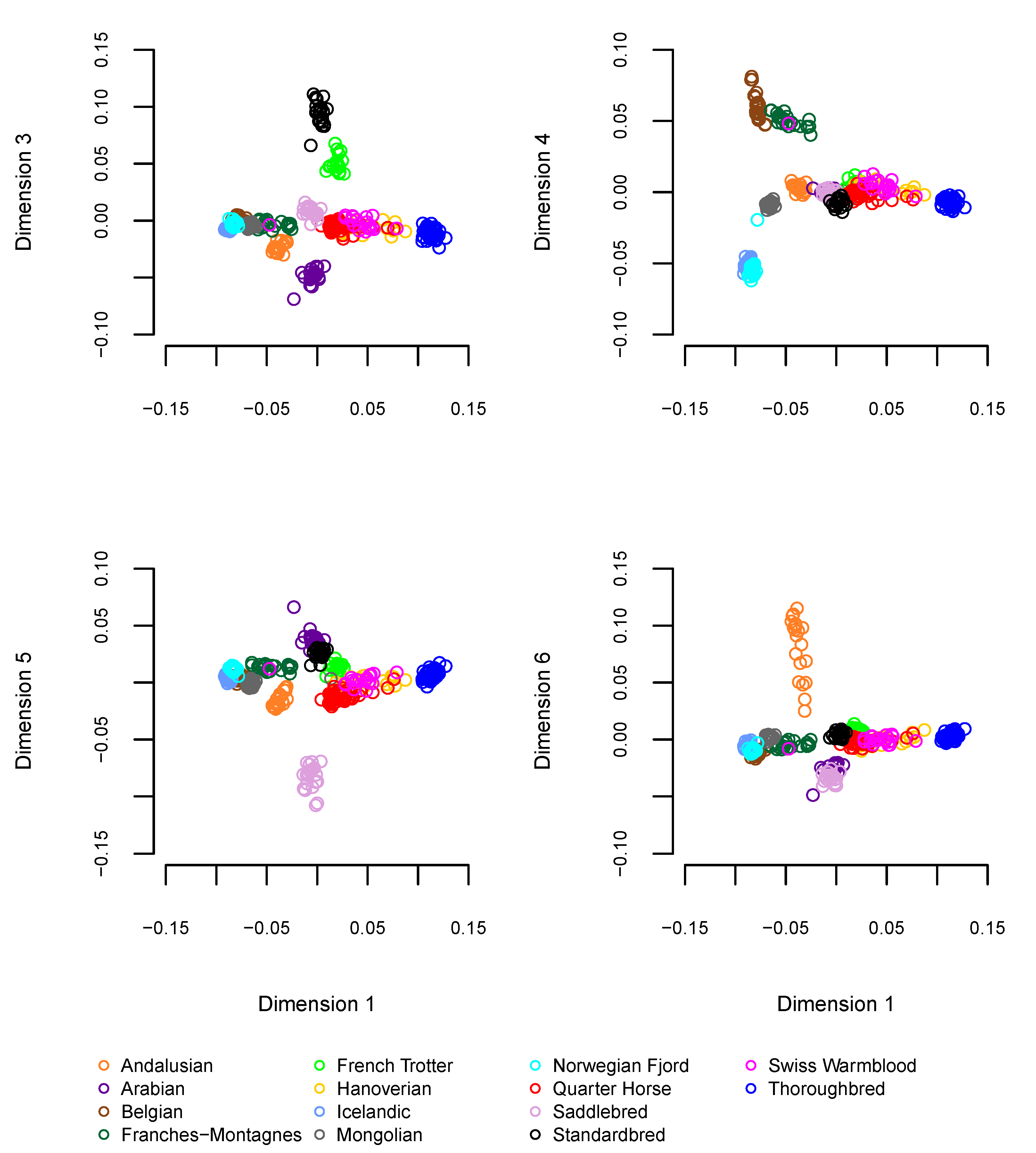

Supplement: Figure S3 — Multidimensional scaling with 14 domestic horse breeds. Metric multidimensional scaling analysis of pair-wise genetic distance was used as described in Materials and Methods to identify relationships between the 14 domestic horse breeds. In these plots dimensions 3–6 (y axes) are always plotted against dimension 1 (x axis). (TIF) [file pgen.1002451.s003.tif]

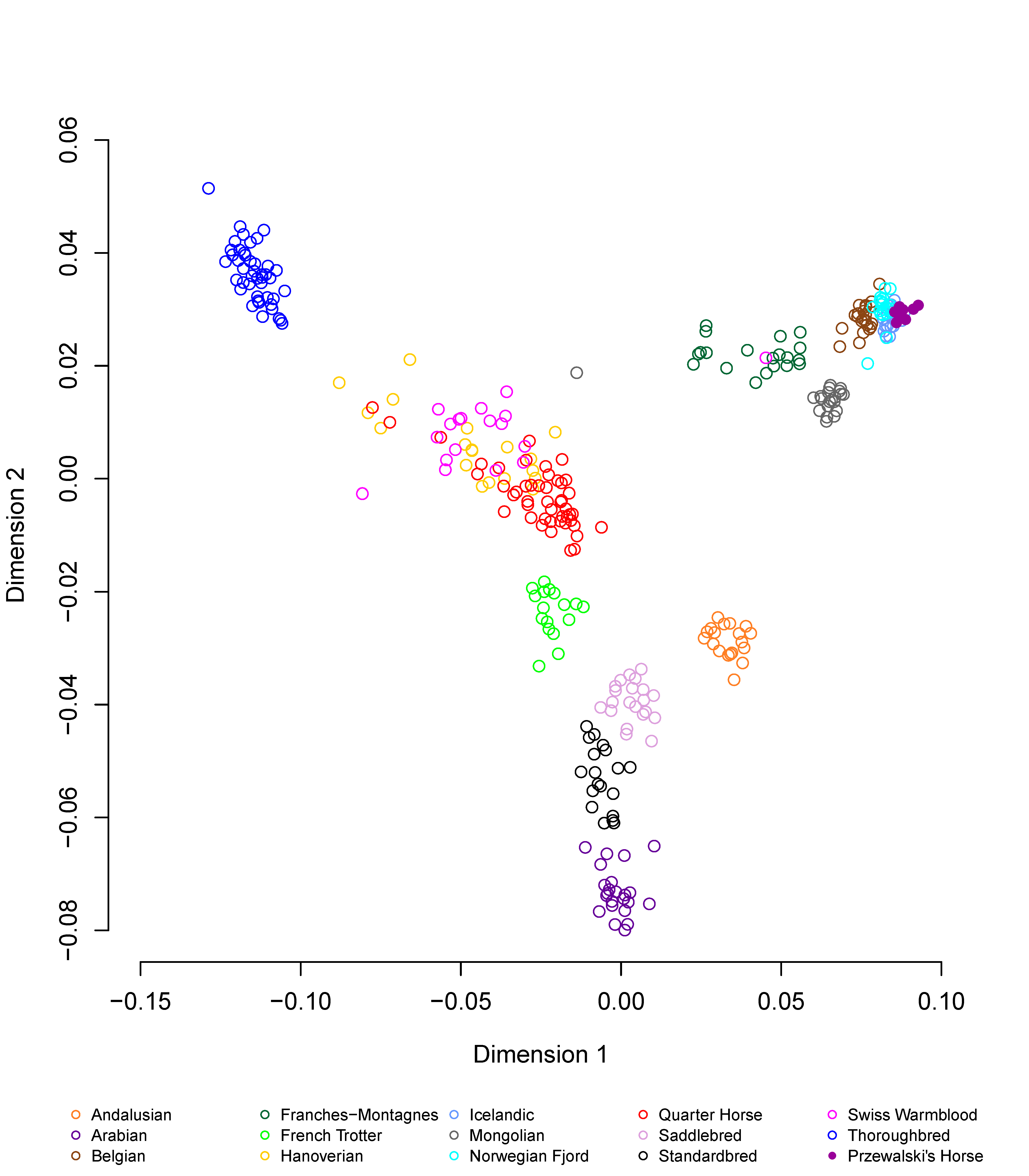

Supplement: Figure S4 — Multidimensional scaling of Przewalski's Horse and domestic horse breeds. Metric multidimensional scaling analysis of pair-wise genetic distance was used as described in Materials and Methods to identify relationships between the 14 domestic breeds and the Przewalski's Horse. (TIF) [file pgen.1002451.s004.tif]

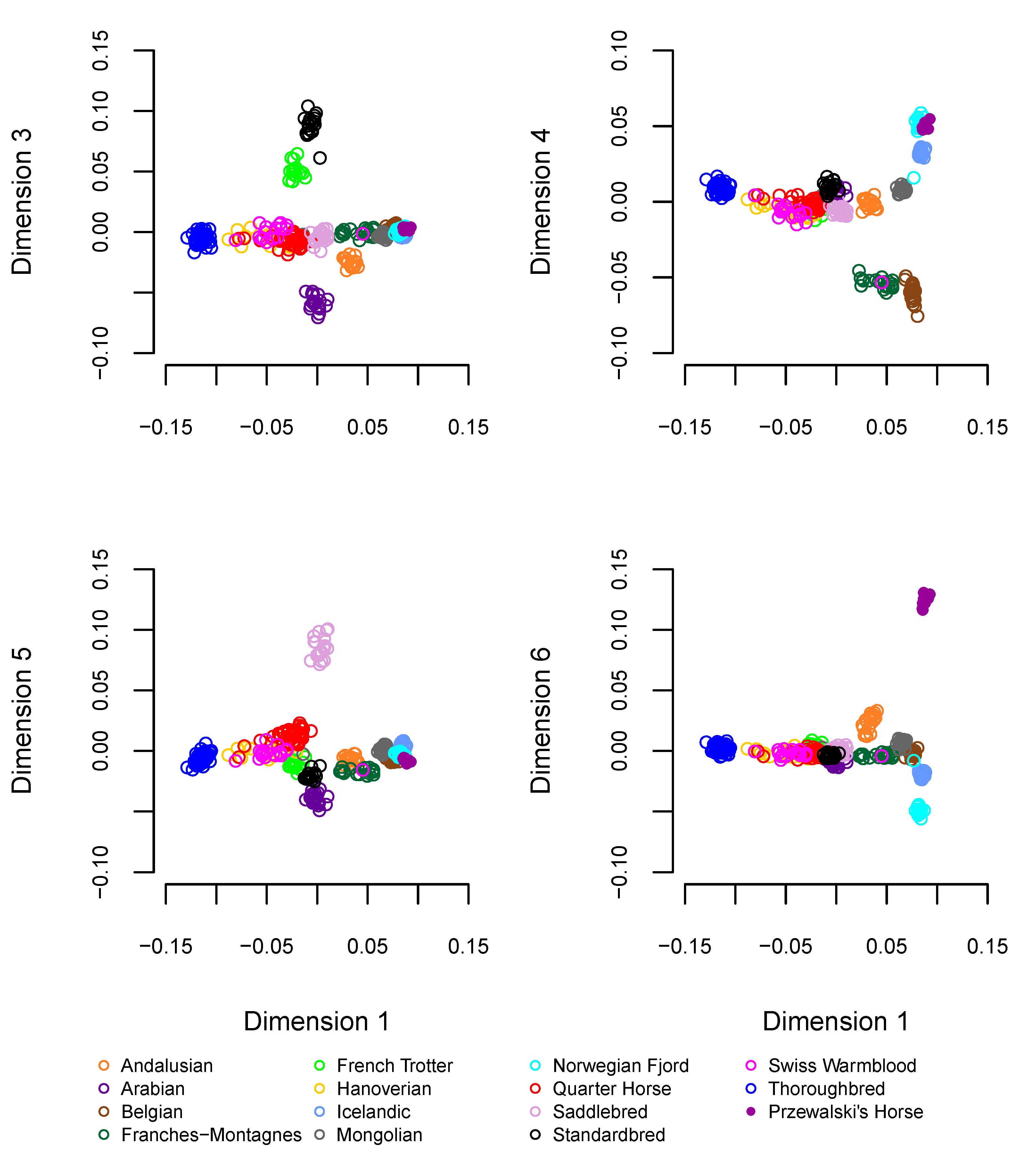

Supplement: Figure S5 — Multidimensional scaling of Przewalski's Horse and domestic horse breeds. Metric multidimensional scaling analysis of pair-wise genetic distance was used as described in Materials and Methods to identify relationships between the 14 domestic breeds and the Przewalski's Horse. In these plots dimensions 3–6 (y axes) are plotted against dimension 1 (x axis). (TIF) [file pgen.1002451.s005.tif]

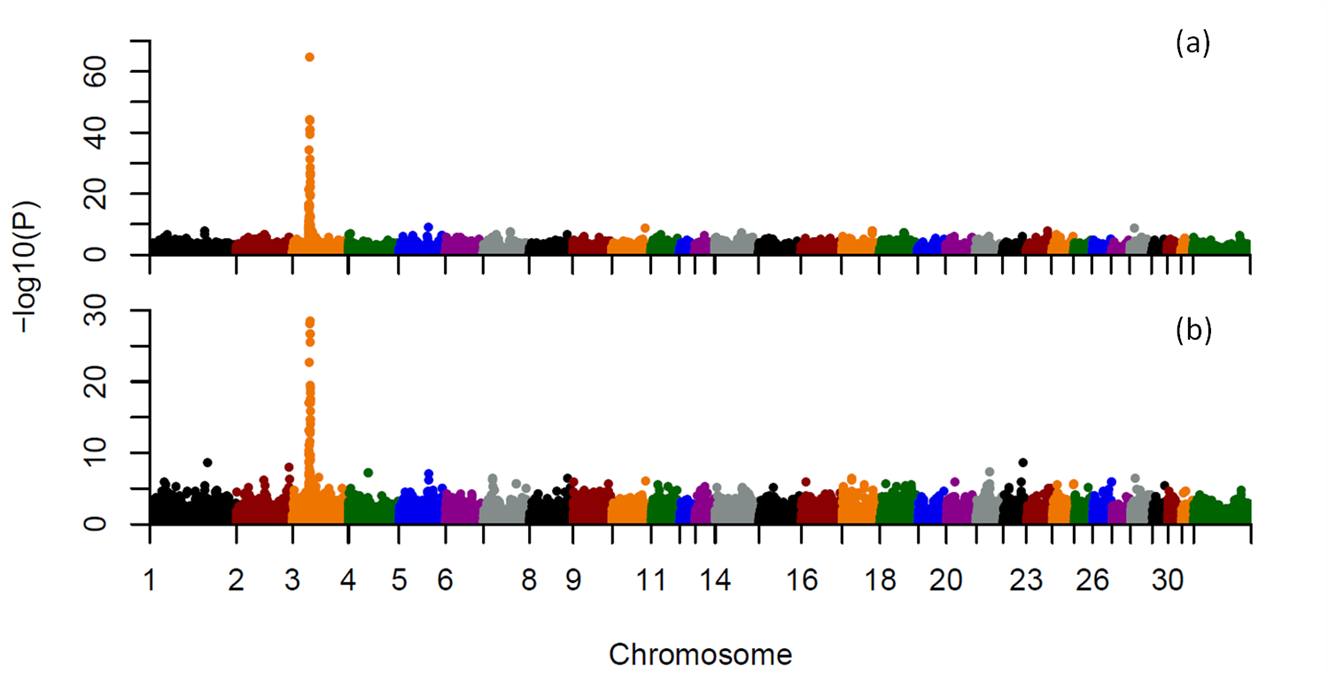

Supplement: Figure S6 — Mapping of the chestnut coat color locus across breeds based on phenotype inferred from all nine genotyped coat color alleles with known interactions (a), or based solely on MC1R genotype and chestnut as a simple recessive trait (b). Phenotypes were inferred as described in Materials and Methods. Unstructured case-control association analyses using chi-square tests for allelic association were then performed on a pruned SNP set also as described in Materials and Methods. SNPs on each chromosome are labeled with a different color on the X axis as indicated. Also indicated on the X axis is the number of pruned SNPs across the genome included in the analysis. (TIF) [file pgen.1002451.s006.tif]

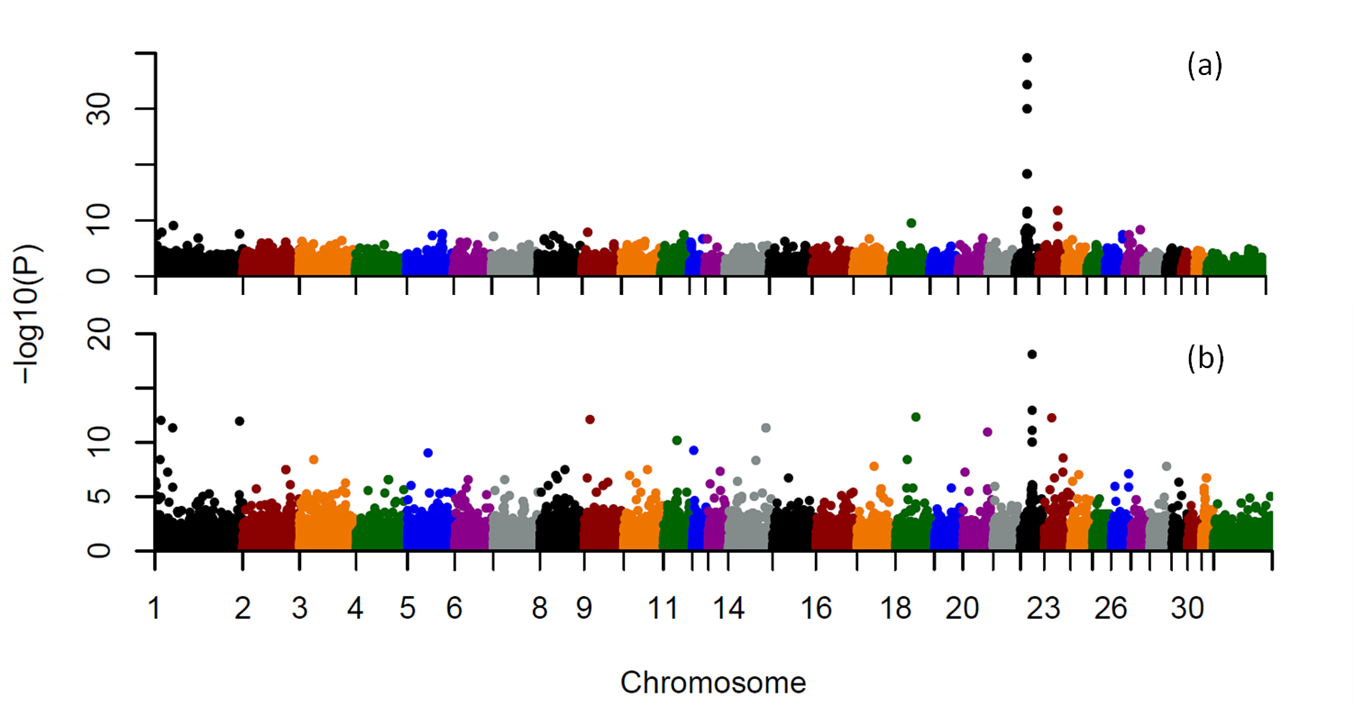

Supplement: Figure S7 — Mapping of the recessive black coat color locus across breeds based on phenotype inferred from all nine genotyped coat color alleles with known interactions (a), or based solely on ASIP genotype and black color as a simple trait (b). Phenotypes were inferred as described in Materials and Methods. Unstructured case-control association analyses using chi-square tests for allelic association were then performed on a pruned SNP set also as described in Materials and Methods. SNPs on each chromosome are labeled with a different color on the X axis as indicated. Also indicated on the X axis is the number of pruned SNPs across the genome included in the analysis. (TIF) [file pgen.1002451.s007.tif]

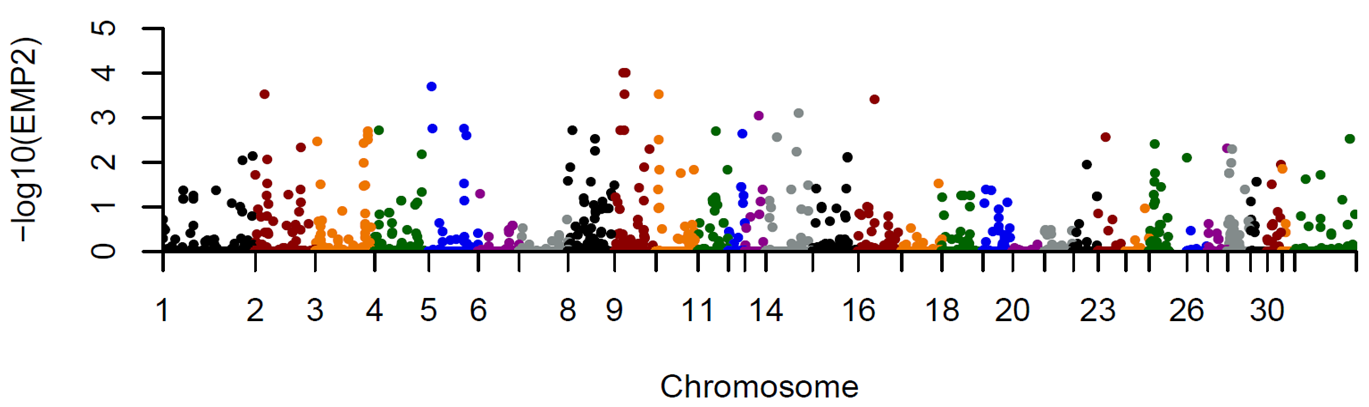

Supplement: Figure S8 — Mapping of the gray coat color locus across breeds based on gray color as a simple dominant trait. Gray phenotype was inferred as described in Materials and Methods. Corrected p-values after 10,000 label-swapping permutations are indicated. SNPs on each chromosome are labeled with a different color on the X axis as indicated. Also indicated on the X axis is the number of pruned SNPs across the genome included in the analysis. (TIF) [file pgen.1002451.s008.tif]

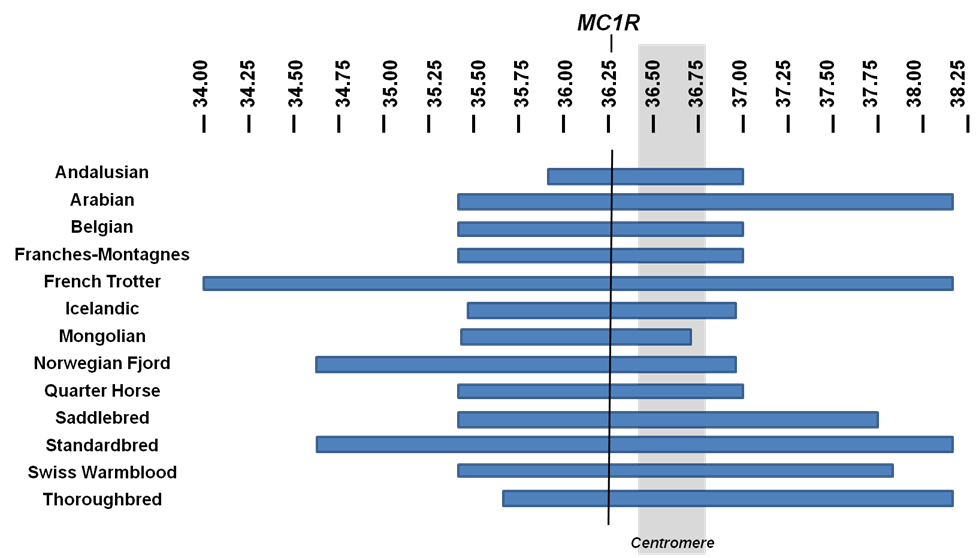

Supplement: Figure S9 — Conserved haplotype at the MC1R locus on ECA3. For all breeds with the exception of the Standardbred, the length of minimal homozygosity for the chestnut allele is depicted. In the Standardbred, where no homozygotes were observed, the shortest length of the haplotype containing the chestnut allele is depicted. The number of chromosomes in each breed that contained the chestnut allele ranged from 7 in the Andalusian, and 10 in the Standardbred and Norwegian Fjord, to 41 in the Saddlebred and Thoroughbred, 48 in the Belgian and 75 in Quarter Horse, with an average of 27.8 across all 14 breeds. The number of SNPs on which the haplotype is based ranged from 16 in the Andalusian to 75 in the Standardbred, with an average of 37.8 across all 14 breeds. The length of the shared haplotype ranged from 1.08 Mb in Andalusian to 4.157 Mb in French Trotter, with an average length of 2.16 Mb across all 14 breeds. The complete list of chromosome numbers, numbers of SNPs on which the haplotype is based, and the length and coordinates of the shared haplotype are available in Table S13. The position of MC1R and the likely position of the centromere are depicted on the figure. Similar analyses resulted in no shared haplotypes for either ASIP or grey. (TIF) [file pgen.1002451.s009.tif]
