# Supplementary material for: A High Density SNP Array for the Domestic Horse and Extant Perissodactyla: Utility for Association Mapping, Genetic Diversity, and Phylogeny Studies
Source: PLoS Genet. 2012 Jan 12;8(1):e1002451. doi: 10.1371/journal.pgen.1002451 (PMC3257288; doi:10.1371/journal.pgen.1002451)
Supplement: Table S1 — Validation rates for the assayed SNPs. The number of selected SNPs from the seven discovery horses and Twilight that provided genotypes (converted), the number of validated SNPs in the equine sample set, and the validation fraction are indicated. SNPs with the highest validation rate were those originally ascertained in two breeds, especially if one of the horses was Twilight. SNPs with the lowest validation rate were originally ascertained in three breeds but were not present in Twilight's sequence. (DOCX) [file pgen.1002451.s010.docx]

**Table S1. Validation rates for the assayed SNPs.** The number of selected SNPs from the seven discovery horses and Twilight that provided genotypes (converted), the number of validated SNPs in the equine sample set, and the validation fraction are indicated. SNPs with the highest validation rate were those originally ascertained in two breeds, especially if one of the horses was Twilight. SNPs with the lowest validation rate were originally ascertained in three breeds but were not present in Twilight’s sequence.

| **Breed of Origin** | **Number Genotyped** | **Number Validated loci** | **Fraction Validated** |
| --- | --- | --- | --- |
| **Akhal Teke** | 7430 | 7291 | 0.981 |
| **Andalusian** | 7406 | 7281 | 0.980 |
| **Arabian** | 6866 | 6714 | 0.978 |
| **Icelandic** | 7182 | 7072 | 0.985 |
| **Quarter Horse** | 6719 | 6564 | 0.978 |
| **Standardbred** | 7106 | 6950 | 0.978 |
| **Thoroughbred (not Twilight)** | 4565 | 4412 | 0.966 |
| **Total SNPs from a single breed** | 47274 | 46284 | 0.979 |
|  |  |  |  |
| **Any two breeds** | 7015 | 6937 | 0.989 |
| **Any three breeds** | 320 | 305 | 0.953 |
| **Any breed and Twilight** | 6170 | 6111 | 0.990 |
| **Any three breeds but not Twilight** | 36 | 32 | 0.889 |
| **Total SNPs from more than 1 breed** | 7328 | 7240 | 0.987 |
| **TOTAL** | 54602 | 53524 | 0.980 |
