# Supplementary material for: A High Density SNP Array for the Domestic Horse and Extant Perissodactyla: Utility for Association Mapping, Genetic Diversity, and Phylogeny Studies
Source: PLoS Genet. 2012 Jan 12;8(1):e1002451. doi: 10.1371/journal.pgen.1002451 (PMC3257288; doi:10.1371/journal.pgen.1002451)
Supplement: Table S2 — Breeds included in the study. Breed, number of individuals, and geographic origin of horses genotyped in this study. *The control horses consisted of the 7 SNP discovery horses listed in Table S1 and the Thoroughbred mare Twilight used to generate the equine genome assembly. (DOCX) [file pgen.1002451.s011.docx]

**Table S2. Breeds included in the study.** Breed, number of individuals, and geographic origin of horses genotyped in this study. *The control horses consisted of the 7 SNP discovery horses listed in Supporting Table 1 and the Thoroughbred mare Twilight used to generate the equine genome assembly.

| **Breed** | **Total genotyped** | **Unrelated** | **Trios** | **Failed to genotype** | **Average genotyping rate (remaining samples)** | **Sample Origin** |
| --- | --- | --- | --- | --- | --- | --- |
| **Andalusian** | 20 | 17 | 1 | 0 | 0.995 | USA |
| **Arabian** | 24 | 24 | 0 | 0 | 0.995 | USA |
| **Belgian** | 24 | 20 | 2 | 0 | 0.995 | USA |
| **Franches-Montagnes** | 23 | 20 | 1 | 1 | 0.996 | Switzerland |
| **French Trotter** | 18 | 15 | 1 | 0 | 0.995 | France |
| **Hanoverian** | 20 | 17 | 1 | 0 | 0.996 | Germany |
| **Icelandic** | 18 | 15 | 1 | 0 | 0.995 | Sweden |
| **Mongolian** | 22 | 22 | 0 | 1 | 0.990 | Japan |
| **Norwegian Fjord** | 22 | 19 | 1 | 0 | 0.995 | Norway |
| **Saddlebred** | 23 | 20 | 1 | 0 | 0.995 | USA |
| **Standardbred** | 20 | 17 | 1 | 0 | 0.996 | Norway |
| **Swiss Warmblood** | 20 | 17 | 1 | 1 | 0.996 | Switzerland |
| **Thoroughbred** | 44 | 31 | 6 | 0 | 0.996 | USA, UK, Ireland |
| **Quarter Horse** | 48 | 45 | 1 | 0 | 0.996 | USA |
| **Controls *** | 8 | n/a | n/a | 0 | 0.996 | Various |
| **Total** | 354 | 299 | 18 | 3 |  |  |
|  |  | (47 individuals) | | |  |  |
