# Supplementary material for: A High Density SNP Array for the Domestic Horse and Extant Perissodactyla: Utility for Association Mapping, Genetic Diversity, and Phylogeny Studies
Source: PLoS Genet. 2012 Jan 12;8(1):e1002451. doi: 10.1371/journal.pgen.1002451 (PMC3257288; doi:10.1371/journal.pgen.1002451)
Supplement: Table S3 — Tests of Mendelian inheritance in trios. Mendelian errors in each of the 18 nuclear trios were calculated and results are reported as Mendelian agreement as described in the Materials and Methods. (DOCX) [file pgen.1002451.s012.docx]

**Table S3. Tests of Mendelian inheritance in trios.** Mendelian errors in each of the 18 nuclear trios were calculated and results are reported as Mendelian agreement as described in the Materials and Methods.

|  | **Correct calls** | **Mendelian errors** | **Total calls** | **Concordant proportion** |
| --- | --- | --- | --- | --- |
| **Andalusian** | 54249 | 0 | 54249 | 1.000 |
| **Belgian-1** | 54078 | 4 | 54082 | 0.999 |
| **Belgian-2** | 54081 | 2 | 54083 | 1.000 |
| **Franches-Montagnes** | 48798 | 5436 | 54234 | 0.899* |
| **French Trotter** | 54254 | 0 | 54254 | 1.000 |
| **Hanoverian** | 54252 | 0 | 54252 | 1.000 |
| **Icelandic** | 54127 | 1 | 54128 | 0.999 |
| **Norwegian Fjord** | 53558 | 469 | 54027 | 0.991** |
| **Quarter Horse** | 54193 | 1 | 54194 | 1.000 |
| **Saddlebred** | 46939 | 7287 | 54226 | 0.867*** |
| **Standardbred** | 54217 | 1 | 54218 | 1.000 |
| **Swiss Warmblood** | 54075 | 0 | 54075 | 1.000 |
| **Thoroughbred-1** | 54230 | 0 | 54230 | 1.000 |
| **Thoroughbred-2** | 54230 | 0 | 54230 | 1.000 |
| **Thoroughbred-3** | 54231 | 0 | 54231 | 1.000 |
| **Thoroughbred-4** | 54166 | 0 | 54166 | 1.000 |
| **Thoroughbred-5** | 54167 | 0 | 54167 | 1.000 |
| **Thoroughbred-6** | 54185 | 0 | 54185 | 1.000 |

* The majority of Mendelian errors in this trio (83.4%) were due to heterozygous genotype calls in the offspring when both parents were homozygous for the same allele.

**All the Mendelian errors for this trio occurred when one parent had no genotype call, the other parent was homozygous for one allele, and the offspring was homozygous for the opposite allele

***The sire and dam of this trio genotyped as the same individual (pi-hat values of 1.0), and the trio was considered invalid due to sample submission error.
