# Supplementary material for: A High Density SNP Array for the Domestic Horse and Extant Perissodactyla: Utility for Association Mapping, Genetic Diversity, and Phylogeny Studies
Source: PLoS Genet. 2012 Jan 12;8(1):e1002451. doi: 10.1371/journal.pgen.1002451 (PMC3257288; doi:10.1371/journal.pgen.1002451)
Supplement: Table S4 — Inter-SNP spacing by chromosome. Polymorphic SNPs were defined as having at least one heterozygous individual (i.e., MAF>0) across all 14 breeds. (DOCX) [file pgen.1002451.s013.docx]

**Table S4. Inter-SNP spacing by chromosome.** Polymorphic SNPs were defined as having at least one heterozygous individual (*i.e.,* MAF > 0) across all 14 breeds.

|  | **Inter-SNP spacing (kb)** | | |  |
| --- | --- | --- | --- | --- |
| **Chromosome** | **Mean** | **Std dev** | **Max** | **Number gaps  > 500kb** |
| **ECA 1** | 42.49 | 52.62 | 496.55 | 0 |
| **ECA 2** | 42.90 | 54.72 | 581.41 | 2 |
| **ECA 3** | 42.42 | 52.68 | 507.83 | 1 |
| **ECA 4** | 42.54 | 55.23 | 504.91 | 1 |
| **ECA 5** | 43.87 | 56.56 | 488.45 | 0 |
| **ECA 6** | 43.97 | 69.98 | 1647.53 | 2 |
| **ECA 7** | 43.94 | 60.66 | 713.36 | 3 |
| **ECA 8** | 43.28 | 54.01 | 398.55 | 0 |
| **ECA 9** | 42.54 | 51.24 | 347.92 | 0 |
| **ECA 10** | 43.79 | 54.65 | 422.66 | 0 |
| **ECA 11** | 42.18 | 53.95 | 593.68 | 0 |
| **ECA 12** | 46.54 | 56.41 | 329.87 | 0 |
| **ECA 13** | 44.28 | 53.98 | 467.12 | 0 |
| **ECA 14** | 42.13 | 54.20 | 444.65 | 0 |
| **ECA 15** | 42.43 | 52.41 | 460.70 | 0 |
| **ECA 16** | 42.03 | 53.19 | 407.51 | 0 |
| **ECA 17** | 43.00 | 56.71 | 462.40 | 0 |
| **ECA 18** | 43.10 | 51.07 | 312.73 | 0 |
| **ECA 19** | 42.27 | 53.91 | 582.53 | 1 |
| **ECA 20** | 43.24 | 53.11 | 333.95 | 0 |
| **ECA 21** | 42.61 | 54.22 | 399.49 | 0 |
| **ECA 22** | 42.25 | 48.86 | 332.06 | 0 |
| **ECA 23** | 44.59 | 54.01 | 397.14 | 0 |
| **ECA 24** | 42.46 | 54.60 | 427.72 | 0 |
| **ECA 25** | 42.69 | 51.89 | 408.45 | 0 |
| **ECA 26** | 43.65 | 52.99 | 364.82 | 0 |
| **ECA 27** | 45.58 | 57.56 | 386.96 | 0 |
| **ECA 28** | 42.34 | 50.63 | 398.71 | 0 |
| **ECA 29** | 43.36 | 58.95 | 622.10 | 1 |
| **ECA 30** | 42.03 | 54.50 | 610.99 | 1 |
| **ECA 31** | 41.65 | 47.82 | 341.28 | 0 |
| **ECA X** | 48.88 | 66.68 | 748.44 | 5 |
