# Supplementary material for: A High Density SNP Array for the Domestic Horse and Extant Perissodactyla: Utility for Association Mapping, Genetic Diversity, and Phylogeny Studies
Source: PLoS Genet. 2012 Jan 12;8(1):e1002451. doi: 10.1371/journal.pgen.1002451 (PMC3257288; doi:10.1371/journal.pgen.1002451)
Supplement: Table S5 — Allele frequencies and genetic diversity in the domestic horse. The mean and median MAF (including all SNPs), the number of polymorphic (MAF≥0.01) and informative SNPs (MAF>0.05), and genetic diversity as determined by heterozygosity (HE), are indicated and calculated as described in Materials and Methods. (DOCX) [file pgen.1002451.s014.docx]

**Table S5. Allele frequencies and genetic diversity in the domestic horse.** The mean and median MAF (including all SNPs), the number of polymorphic (MAF ≥ 0.01) and informative SNPs (MAF > 0.05), and genetic diversity as determined by heterozygosity (H_E_), are indicated and calculated as described in Materials and Methods.

|  | **Mean MAF** | **Median MAF** | **Total polymorphic SNPs (proportion of polymorphic SNPs)** | **Informative SNPS (proportion of informative SNPs)** | **Gene diversity**  **H_E_** |
| --- | --- | --- | --- | --- | --- |
| **All breeds** | 0.236 | 0.224 | 53,066 (0.99) | 49,603 (0.91) | N/A |
| **Andalusian** | 0.206 | 0.200 | 47,807 (0.89) | 41,218 (0.75) | 0.284 |
| **Arabian** | 0.211 | 0.208 | 47,374 (0.89) | 42,140 (0.77) | 0.275 |
| **Belgian** | 0.182 | 0.146 | 44,372 (0.83) | 37,808 (0.69) | 0.266 |
| **Franches-Montagnes** | 0.197 | 0.182 | 47,279 (0.88) | 40,868 (0.75) | 0.271 |
| **French Trotter** | 0.207 | 0.194 | 47,120 (0.88) | 43,605 (0.80) | 0.262 |
| **Hanoverian** | 0.229 | 0.225 | 49,386 (0.92) | 44,441 (0.81) | 0.275 |
| **Icelandic** | 0.184 | 0.167 | 43,287 (0.81) | 39,534 (0.72) | 0.275 |
| **Mongolian** | 0.201 | 0.167 | 50,079 (0.94) | 42,633 (0.78) | 0.292 |
| **Norwegian Fjord** | 0.180 | 0.159 | 43,530 (0.81) | 37,053 (0.68) | 0.265 |
| **Quarter Horse** | 0.232 | 0.229 | 52,085 (0.97) | 47,699 (0.87) | 0.287 |
| **Saddlebred** | 0.206 | 0.196 | 47,646 (0.89) | 41,657 (0.76) | 0.268 |
| **Standardbred** | 0.200 | 0.175 | 45,916 (0.86) | 39,379 (0.72) | 0.261 |
| **Swiss Warmblood** | 0.230 | 0.237 | 49,827 (0.93) | 47,121 (0.86) | 0.281 |
| **Thoroughbred** | 0.224 | 0.216 | 47,701 (0.89) | 43,343 (0.79) | 0.247 |
