# Supplementary material for: A High Density SNP Array for the Domestic Horse and Extant Perissodactyla: Utility for Association Mapping, Genetic Diversity, and Phylogeny Studies
Source: PLoS Genet. 2012 Jan 12;8(1):e1002451. doi: 10.1371/journal.pgen.1002451 (PMC3257288; doi:10.1371/journal.pgen.1002451)
Supplement: Table S6 — Proportion of validated SNPs from each discovery breed across all breeds genotyped. The impact of discovery breed on SNP validation rate across the genotyped sample set was determined as described in Materials and Methods. (DOCX) [file pgen.1002451.s015.docx]

**Table S6. Proportion of validated SNPs from each discovery breed across all breeds genotyped.** The impact of discovery breed on SNP validation rate across the genotyped sample set was determined as described in Materials and Methods.

|  |  | **Discovery Breed** | | | | | | |
| --- | --- | --- | --- | --- | --- | --- | --- | --- |
|  |  | **Akhal-Teke** | **Andalusian** | **Arabian** | **Icelandic** | **Quarter Horse** | **Standardbred** | **Thoroughbred** |
| **Genotyped Breed** | **Standardbred** | 0.704 | 0.686 | 0.710 | 0.662 | 0.712 | 0.757 | 0.726 |
|  | **Thoroughbred** | 0.826 | 0.713 | 0.748 | 0.668 | 0.785 | 0.742 | 0.855 |
|  | **Swiss Warmblood** | 0.825 | 0.817 | 0.8417 | 0.789 | 0.842 | 0.830 | 0.861 |
|  | **Quarter Horse** | 0.825 | 0.816 | 0.835 | 0.784 | 0.852 | 0.835 | 0.856 |
|  | **Norwegian Fjord** | 0.659 | 0.669 | 0.663 | 0.680 | 0.668 | 0.673 | 0.660 |
|  | **Icelandic** | 0.780 | 0.784 | 0.777 | 0.775 | 0.773 | 0.774 | 0.767 |
|  | **Hanoverian** | 0.771 | 0.759 | 0.794 | 0.720 | 0.799 | 0.776 | 0.835 |
|  | **French Trotter** | 0.780 | 0.769 | 0.793 | 0.740 | 0.797 | 0.795 | 0.813 |
|  | **Frances-Montagnes** | 0.736 | 0.738 | 0.735 | 0.716 | 0.749 | 0.738 | 0.749 |
|  | **Belgian** | 0.679 | 0.692 | 0.678 | 0.680 | 0.682 | 0.687 | 0.676 |
|  | **Arabian** | 0.741 | 0.723 | 0.823 | 0.688 | 0.743 | 0.730 | 0.750 |
|  | **Andalusian** | 0.733 | 0.811 | 0.748 | 0.698 | 0.734 | 0.730 | 0.741 |
|  | **ALL** | 0.862 | 0.865 | 0.877 | 0.851 | 0.870 | 0.867 | 0.874 |
