# Supplementary material for: A High Density SNP Array for the Domestic Horse and Extant Perissodactyla: Utility for Association Mapping, Genetic Diversity, and Phylogeny Studies
Source: PLoS Genet. 2012 Jan 12;8(1):e1002451. doi: 10.1371/journal.pgen.1002451 (PMC3257288; doi:10.1371/journal.pgen.1002451)
Supplement: Table S7 — Species, genotyping rates, sample and locus GC scores for unfiltered data in extant Perissodactyla. Genotyping rates and GC scores reported using Illumina's default calling parameters as described in Materials and Methods. (DOCX) [file pgen.1002451.s016.docx]

**Table S7. Species, genotyping rates, sample and locus GC scores for unfiltered data in extant Perissodactyla.**

| **Species** | **N** | **Loci producing a genotype ^b^** | **Mean individual genotyping rate** | **Mean GC10 ^c^** |
| --- | --- | --- | --- | --- |
| **Przewalski's Horse**  *Equus przewalskii* | 9 | 54410 | 0.992 | 0.724 |
| **Domestic Ass**  *Equus asinus* | 2 | 51602 | 0.939 | 0.704 |
| **Somali Wild Ass**  *Equus asinus somalicus* | 3 | 51707 | 0.939 | 0.699 |
| **Persian Onager**  *Equus hemionus onager* | 4 | 52017 | 0.943 | 0.705 |
| **Transcaspian Kulan**  *Equus hemionus kulan* | 3 | 52014 | 0.944 | 0.706 |
| **Eastern Kiang**  *Equus kiang holdereri* | 3 | 51786 | 0.942 | 0.707 |
| **Grant's Zebra**  *Equus quagga boehmi* | 2 | 51833 | 0.943 | 0.704 |
| **Grevy's Zebra**  *Equus grevyi* | 3 | 51797 | 0.944 | 0.707 |
| **Hartmann's Mountain Zebra**  *Equus zebra hartmannae* | 4^a^ | 51719 | 0.935 | 0.695 |
| **Malayan Tapir**  *Tapirus indicus indicus* | 2 | 12964 | 0.195 | 0.240 |
| **Tapir**  *Tapirus bairdii* | 2 | 12758 | 0.185 | 0.228 |
| **Mountain Tapir**  *Tapirus pinchaque* | 3 | 12716 | 0.199 | 0.251 |
| **East African Black Rhino**  *Diceros bicornis michaeli* | 2 | 13609 | 0.206 | 0.236 |
| **South African Black Rhino**  *Diceros bicornis minor* | 2 | 10661 | 0.195 | 0.233 |
| **Southern White Rhino**  *Ceratotherium simum simum* | 2 | 13552 | 0.196 | 0.219 |
| **Sumatran Rhino**  *Dicerorhinus sumatrensis* | 3 | 13277 | 0.186 | 0.231 |
| **Northern White Rhino**  *Ceratotherium simum cottoni* | 2 | 13376 | 0.204 | 0.236 |
| **Great Indian Rhino**  *Rhinoceros unicornis* | 2 | 20461 | 0.375 | 0.253 |
| **Domestic Horse** | 351 | 54602 | 0.996 | 0.730 |
| **All Hippomorpha** | 32 | n/a | 0.959 | 0.705 |
| **All Ceratomorpha** | 20 | n/a | 0.246 | 0.236 |

^a^ one individual completely failed to genotype. Data in the table is calculated from the remaining three individuals

^b^ the number of loci that produced genotypes across all the individuals

^c^ mean Illumina GenCall 10 (GC10) score across all loci producing a genotype in each species
