# Supplementary material for: A High Density SNP Array for the Domestic Horse and Extant Perissodactyla: Utility for Association Mapping, Genetic Diversity, and Phylogeny Studies
Source: PLoS Genet. 2012 Jan 12;8(1):e1002451. doi: 10.1371/journal.pgen.1002451 (PMC3257288; doi:10.1371/journal.pgen.1002451)
Supplement: Table S8 — Quality scores, SNP conversion and SNP validation rates and mean heterozygosity for extant Perissodactyla after filtering for intensity and genotyping rate. Genotyping rates, conversion and validation rates and observed hetrozygosities after filtering the data as described in Materials and Methods. Conversion and validation rates and heterozygosities are not reported for Ceratomorpha due to low genotyping quality. (DOCX) [file pgen.1002451.s017.docx]

**Table S8. Quality scores, SNP conversion and SNP validation rates for extant Perissodactyla after filtering for intensity and genotyping rate.**

| **Species** | **Loci producing a genotype ^b^** | **Number of individuals** | **Loci with genotyping rates >90%** | **Mean GC10 score** | **SNP conversion rate^c^** | **Validated loci^d^** | **SNP validation rate^e^** | **Average Obs Het** |
| --- | --- | --- | --- | --- | --- | --- | --- | --- |
| **Przewalski's Horse**  *Equus przewalskii* | 54410 | 8 | 53473 | 0.737 | 0.891 | 26859 | 0.508 | 0.168 |
| **Domestic Ass**  *Equus asinus* | 51602 | 2 | 50965 | 0.717 | 0.849 | 299 | 0.007 | 0.003 |
| **Somali Wild Ass**  *Equus asinus somalicus* | 51662 | 2 | 51132 | 0.719 | 0.852 | 265 | 0.008 | 0.003 |
| **Persian Onager**  *Equus hemionus onager* | 52015 | 4 | 50598 | 0.722 | 0.843 | 791 | 0.018 | 0.006 |
| **Transcaspian Kulan**  *Equus hemionus kulan* | 51964 | 2 | 51393 | 0.721 | 0.857 | 595 | 0.012 | 0.006 |
| **Eastern Kiang**  *Equus kiang holdereri* | 51786 | 3 | 51010 | 0.721 | 0.850 | 397 | 0.010 | 0.004 |
| **Grant's Zebra**  *Equus quagga boehmi* | 51833 | 2 | 51167 | 0.716 | .0853 | 644 | 0.014 | 0.007 |
| **Grevy's Zebra**  *Equus grevyi* | 51797 | 3 | 51179 | 0.723 | 0.853 | 364 | 0.009 | 0.003 |
| **Hartmann's Mountain Zebra**  *Equus zebra hartmannae* | 51690 | 4 | 50044 | 0.712 | 0.834 | 321 | 0.009 | 0.003 |
| **Malayan Tapir**  *Tapirus indicus indicus* | 9783 | 2 | 7102 | 0.408 | n/a | n/a | n/a | n/a |
| **Tapir**  *Tapirus bairdii* | 8358 | 2 | 5509 | 0.451 | n/a | n/a | n/a | n/a |
| **Mountain Tapir**  *Tapirus pinchaque* | 10286 | 3 | 6289 | 0.472 | n/a | n/a | n/a | n/a |
| **East African Black Rhino**  *Diceros bicornis michaeli* | 9648 | 2 | 6370 | 0.429 | n/a | n/a | n/a | n/a |
| **South African Black Rhino**  *Diceros bicornis minor* | 8038 | 1 | n/a | 0.200 | n/a | n/a | n/a | n/a. |
| **Southern White Rhino**  *Ceratotherium simum simum* | 8094 | 2 | 5878 | 0.439 | n/a | n/a | n/a | n/a |
| **Sumatran Rhino**  *Dicerorhinus sumatrensis* | 8849 | 3 | 5840 | 0.467 | n/a | n/a | n/a | n/a |
| **Northern White Rhino**  *Ceratotherium simum cottoni* | 9241 | 2 | 7038 | 0.432 | n/a | n/a | n/a | n/a |
| **Great Indian Rhino**  *Rhinoceros unicornis* | 86 | 0 | n/a | n/a | n/a | n/a | n/a | n/a |

^a^ individual genotyping rate is defined as the proportion of SNP that produced a genotype with that individual

^b^ the number of loci that produced genotypes across all the individuals

^c^ SNP genotyping rate calculated as number of SNPs that produced a genotype/ total SNPs

^d^ loci with at least one heterozygous call

^e^ proportion of converted SNPs that were polymorphic
