# Supplementary material for: A High Density SNP Array for the Domestic Horse and Extant Perissodactyla: Utility for Association Mapping, Genetic Diversity, and Phylogeny Studies
Source: PLoS Genet. 2012 Jan 12;8(1):e1002451. doi: 10.1371/journal.pgen.1002451 (PMC3257288; doi:10.1371/journal.pgen.1002451)
Supplement: Table S9 — Inbreeding coefficients in each breed. Individual inbreeding coefficients (F) were estimated with 17,947 SNPs that were pruned for linkage equilibrium as described in Materials and Methods. (DOCX) [file pgen.1002451.s018.docx]

**Table S9. Inbreeding coefficients in each breed.** Individual inbreeding coefficients (F) were estimated with 17,947 SNPs that were pruned for linkage equilibrium as described in Materials and Methods.

| **Breed** | **Mean of individual inbreeding coefficients (F)** | **Standard deviation** |
| --- | --- | --- |
| **Thoroughbred** | 0.15 | 0.02 |
| **Standardbred** | 0.12 | 0.04 |
| **Norwegian Fjord** | 0.11 | 0.04 |
| **French trotter** | 0.11 | 0.03 |
| **Arabian** | 0.10 | 0.05 |
| **Belgian** | 0.10 | 0.02 |
| **Saddlebred** | 0.10 | 0.03 |
| **Andalusian** | 0.09 | 0.07 |
| **Swiss Warmblood** | 0.09 | 0.04 |
| **Franches-Montagnes** | 0.09 | 0.04 |
| **Icelandic** | 0.09 | 0.04 |
| **Hanoverian** | 0.06 | 0.02 |
| **Quarter Horse** | 0.04 | 0.03 |
| **Mongolian** | 0.02 | 0.01 |
