# Supplementary material for: A High Density SNP Array for the Domestic Horse and Extant Perissodactyla: Utility for Association Mapping, Genetic Diversity, and Phylogeny Studies
Source: PLoS Genet. 2012 Jan 12;8(1):e1002451. doi: 10.1371/journal.pgen.1002451 (PMC3257288; doi:10.1371/journal.pgen.1002451)
Supplement: Table S10 — Mean pair-wise genetic distances in domestic horse populations. Genetic distance (D) between pair-wise combinations of individuals was calculated as described in Materials and Methods. (DOCX) [file pgen.1002451.s019.docx]

**Table S10. Mean pair-wise genetic distances in domestic horse populations.** Genetic distance (D) between pair-wise combinations of individuals was calculated as described in Materials and Methods.

|  |  | **Genetic Distances** | |
| --- | --- | --- | --- |
| **Population** | **Number of pairs** | **D** | **Standard deviation** |
| **Across breeds** | 49049 | 0.27 | 0.014 |
| **Within breeds** | 4252 | 0.24 | 0.020 |
| **Quarter Horse** | 1081 | 0.26 | 0.006 |
| **Swiss Warmblood** | 136 | 0.26 | 0.014 |
| **Hanoverian** | 171 | 0.25 | 0.015 |
| **Andalusian** | 171 | 0.24 | 0.018 |
| **Thoroughbred** | 946 | 0.24 | 0.014 |
| **Belgian** | 253 | 0.23 | 0.011 |
| **Arabian** | 253 | 0.23 | 0.009 |
| **French Trotter** | 136 | 0.23 | 0.008 |
| **Mongolian** | 210 | 0.23 | 0.013 |
| **Saddlebred** | 210 | 0.23 | 0.008 |
| **Franches-Montagnes** | 190 | 0.22 | 0.087 |
| **Standardbred** | 171 | 0.22 | 0.010 |
| **Icelandic** | 136 | 0.21 | 0.008 |
| **Norwegian Fjord** | 210 | 0.21 | 0.013 |
