# Supplementary material for: A High Density SNP Array for the Domestic Horse and Extant Perissodactyla: Utility for Association Mapping, Genetic Diversity, and Phylogeny Studies
Source: PLoS Genet. 2012 Jan 12;8(1):e1002451. doi: 10.1371/journal.pgen.1002451 (PMC3257288; doi:10.1371/journal.pgen.1002451)
Supplement: Table S11 — Mean pair-wise distances between Przewalski's Horse and domestic horse populations. Genetic distance (D) between pair-wise combinations of individuals was calculated as described in Materials and Methods. (DOCX) [file pgen.1002451.s020.docx]

**Table S11. Mean pair-wise distances between Przewalski’s Horse and domestic horse populations.** Genetic distance (D) between pair-wise combinations of individuals was calculated as described in Materials and Methods.

|  |  | **Genetic Distances** | |
| --- | --- | --- | --- |
| **Population** | **Number of pairs** | **D** | **Standard deviation** |
| **Within Prezwalski's Horse** | 36 | 0.15 | 0.021 |
| **Przewalski's & Domestic Horse** | 2943 | 0.28 | 0.021 |
|  |  |  |  |
| **Mongolian-Przewalski** | 189 | 0.25 | 0.007 |
| **Norwegian Fjord-Przewalski** | 189 | 0.25 | 0.004 |
| **Belgian-Przewalski** | 198 | 0.25 | 0.004 |
| **Icelandic-Przewalski** | 153 | 0.25 | 0.004 |
| **Franches-Montagnes-Przewalski** | 180 | 0.26 | 0.005 |
| **Andalusian-Przewalski** | 171 | 0.27 | 0.005 |
| **Saddlebred-Przewalski** | 198 | 0.28 | 0.004 |
| **Standardbred-Przewalski** | 171 | 0.28 | 0.005 |
| **Quarter Horse-Przewalski** | 423 | 0.28 | 0.007 |
| **French Trotter-Przewalski** | 153 | 0.28 | 0.005 |
| **Arabian-Przewalski** | 207 | 0.28 | 0.004 |
| **Hanoverian-Przewalski** | 171 | 0.29 | 0.008 |
| **Swiss Warmblood-Przewalski** | 153 | 0.29 | 0.007 |
| **Thoroughbred-Przewalski** | 396 | 0.31 | 0.005 |
