# Supplementary material for: A High Density SNP Array for the Domestic Horse and Extant Perissodactyla: Utility for Association Mapping, Genetic Diversity, and Phylogeny Studies
Source: PLoS Genet. 2012 Jan 12;8(1):e1002451. doi: 10.1371/journal.pgen.1002451 (PMC3257288; doi:10.1371/journal.pgen.1002451)
Supplement: Table S12 — Chi square analysis for mapping of known coat color loci across the 14 breeds. Chestnut, black and gray phenotypes were either inferred from the genotypes at 9 known coat color loci, or from the genotype of a single locus (designated as MC1R and ASIP) only, using known inheritance models as described in Materials and Methods. Case control association analyses were then performed on a pruned SNP set also as described in Materials and Methods. The genomic inflation factor lambda, the number of SNPs with an EMP2<0.05 after 10000 label-swapping permutations, the number of these SNPs within 5 Mb of the true locus (true positive SNPs), the length of the chromosomal segment at the true gene locus containing true positive SNPs, and the false discovery rate (percentage of all positive SNPs that are within 5 Mb of the true locus), are all indicated. (DOC) [file pgen.1002451.s021.doc]

**Table S12. Chi square analysis for mapping of known coat color loci across the 14 breeds.**

|  |  |  | **chi-square** | | | | |
| --- | --- | --- | --- | --- | --- | --- | --- |
| **Phenotype** | **n cases** | **n controls** | **Lambda** | **SNPs with EMP2 <0.05** | **Number of true positive SNPs** | **Extent of associated chromosomal segment** | **False discovery rate** |
| Chestnut | 105 | 218 | 1.90619 | 48 | 40 | 21.6 Mb | 0.17 |
| *MC1R* | 127 | 204 | 2.44605 | 98 | 68 | 9.62 Mb | 0.31 |
| Black | 28 | 295 | 2.06633 | 23 | 5 | 0.165 Mb | 0.78 |
| *ASIP* | 55 | 276 | 2.1935 | 35 | 15 | 12.8 Mb | 0.57 |
| Gray | 28 | 310 | 2.36772 | 46 | 1 | single SNP 1.03 Mb from mutation | 0.98 |

Chestnut, black and gray phenotypes were either inferred from the genotypes at 9 known coat color loci, or from the genotype of a single locus (designated as *MC1R* and *ASIP*) only, using known inheritance models as described in Materials and Methods. Case control association analyses were then performed on a pruned SNP set also as described in Materials and Methods. The genomic inflation factor lambda, the number of SNPs with an EMP2 < 0.05 after 10000 label-swapping permutations, the number of these SNPs within 5 Mb of the true locus (true positive SNPs), the length of the chromosomal segment at the true gene locus containing true positive SNPs, and the false discovery rate (percentage of all positive SNPs that are within 5 Mb of the true locus), are all indicated.

Chestnut =chestnut phenotype across all color loci

MC1R =chestnut based on MC1R genotype

Black =black phenotype across all color loci

ASIP =black based on ASIP genotype

Gray =gray phenotype across all color loci
