# Supplementary material for: A High Density SNP Array for the Domestic Horse and Extant Perissodactyla: Utility for Association Mapping, Genetic Diversity, and Phylogeny Studies
Source: PLoS Genet. 2012 Jan 12;8(1):e1002451. doi: 10.1371/journal.pgen.1002451 (PMC3257288; doi:10.1371/journal.pgen.1002451)
Supplement: Table S13 — CMH analysis for mapping of known coat color loci across the 14 breeds. Phenotypes were inferred from multi or single locus genotypes as described in Table S1 and the Materials and Methods. Stratified genome-wide association analysis was performed using the Cochran-Mantel-Haenszel (CMH) test. Horses were clustered for this test on the basis of the pair-wise population concordance test also as described in Materials and Methods. The genomic inflation factor lambda, the number of SNPs with an EMP2<0.05 after 10000 label-swapping permutations, the number of these SNPs within 5 Mb of the true locus (true positive SNPs), the length of the chromosomal segment at the true gene locus containing true positive SNPs, and the false discovery rate (percentage of all positive SNPs that are within 5 Mb of the true locus), are all indicated. (DOC) [file pgen.1002451.s022.doc]

**Table S13. CMH analysis for mapping of known coat color loci across the 14 breeds.**

|  |  |  | **Cochran-Mantel- Haenszel** | | | |
| --- | --- | --- | --- | --- | --- | --- |
| **Phenotype** | **n cases** | **n controls** | **SNPs with EMP2 <0.05** | **Number of true positive SNPs** | **Extent of associated chromosomal segment** | **False discovery rate** |
| Chestnut | 105 | 218 | 39 | 39 | 3.69 Mb | 0.00 |
| *MC1R* | 127 | 204 | 59 | 59 | 4.27 Mb | 0 |
| Black | 28 | 295 | 2 | 2 | SNPs at 0.3 and 0.4 Mb from mutation | 0.00 |
| *ASIP* | 55 | 276 | 4 | 4 | positive SNPs at 24.7 to 25.1, mutation at 25.17 | 0.00 |
| Gray | 28 | 310 | 0 | N/A | N/A | N/A |

Phenotypes were inferred from multi or single locus genotypes as described in Table 1 and the Materials and Methods. Stratified genome-wide association analysis was performed using the Cochran-Mantel- Haenszel (CMH) test. Horses were clustered for this test on the basis of the pair-wise population concordance test also as described in Materials and Methods. The genomic inflation factor lambda, the number of SNPs with an EMP2 < 0.05 after 10000 label-swapping permutations, the number of these SNPs within 5 Mb of the true locus (true positive SNPs), the length of the chromosomal segment at the true gene locus containing true positive SNPs, and the false discovery rate (percentage of all positive SNPs that are within 5 Mb of the true locus), are all indicated.

Chestnut =chestnut phenotype across all color loci

Black =black phenotype across all color loci

MC1R =chestnut based on MC1R genotype

ASIP =black based on ASIP genotype

Gray =gray phenotype across all color loci
