# Supplementary material for: A High Density SNP Array for the Domestic Horse and Extant Perissodactyla: Utility for Association Mapping, Genetic Diversity, and Phylogeny Studies
Source: PLoS Genet. 2012 Jan 12;8(1):e1002451. doi: 10.1371/journal.pgen.1002451 (PMC3257288; doi:10.1371/journal.pgen.1002451)
Supplement: Table S14 — Chi-square tests of association for coat color loci within breeds. Chestnut, black and gray phenotypes were either inferred from the genotypes at 8 known coat color loci, or from the genotype of a single locus (designated as MC1R and ASIP) only, using known inheritance models as described in Materials and Methods. Case-control association analyses were then performed on a pruned SNP set also as described in Materials and Methods. The genomic inflation factor lambda, the number of SNPs with an EMP2<0.05 after 10000 label-swapping permutations, the number of these SNPs within 5 Mb of the true locus, the lowest EMP2 value (if <1.0), and the chromosomal location of the SNP with the lowest EMP2 value, are all indicated. (DOC) [file pgen.1002451.s023.doc]

**Table S14. Chi-square tests of association for coat color loci within breeds.** Chestnut, black and gray phenotypes were either inferred from the genotypes at 8 known coat color loci, or from the genotype of a single locus (designated as *MC1R* and *ASIP*) only, using known inheritance models as described in Materials and Methods. Case-control association analyses were then performed on a pruned SNP set also as described in Materials and Methods. The genomic inflation factor lambda, the number of SNPs with an EMP2 < 0.05 after 10000 label-swapping permutations, the number of these SNPs within 5 Mb of the true locus, the lowest EMP2 value (if < 1.0), and the chromosomal location of the SNP with the lowest EMP2 value, are all indicated.

| **Phenotype (known locus)** | **Breed** | **#cases** | **#controls** | **lambda** | **SNPs with EMP2 <0.05** | **#true positive SNPs** | **lowest EMP2 value** | **location(s) of lowest EMP2** |
| --- | --- | --- | --- | --- | --- | --- | --- | --- |
| Chestnut (ECA3) | Arabian | 7 | 17 | 1.000 | 0 | 0 | N/A | N/A |
|  | Hanoverian | 7 | 12 | 1.314 | 0 | 0 | 0.50 | ECA8 |
|  | Quarter Horse | 22 | 24 | 1.147 | 3 | 3 | 0.01 | ECA3 |
|  | Saddlebred | 13 | 18 | 1.218 | 0 | 0 | 0.27 | ECA3 |
|  | Swiss Warmblood | 8 | 11 | 1.316 | 0 | 0 | 0.40 | ECA9 |
|  | Thoroughbred | 11 | 26 | 1.131 | 4 | 4 | 0.02 | ECA3 |
| *MC1R* (ECA3) | Arabian | 14 | 10 | 1.268 | 1 | 1 | 0.006 | ECA3 |
|  | Hanoverian | 7 | 12 | 1.314 | 0 | 0 | 0.47 | ECA8 |
|  | Mongolian | 7 | 14 | 1.123 | 3 | 3 | 0.65 | ECA5 |
|  | Quarter Horse | 29 | 18 | 1.028 | 7 | 7 | >0.0001 | ECA3 |
|  | Swiss Warmblood | 8 | 11 | 1.316 | 0 | 0 | 0.41 | ECA9 |
|  | Thoroughbred | 10 | 26 | 1.110 | 7 | 7 | 0.006 | ECA3 |
| Black (ECA22) | Icelandic | 8 | 8 | 1.276 | 0 | 0 | 0.69 | ECA10 |
| *ASIP* (ECA22) | Andalusian | 6 | 10 | 1.484 | 3 | 3 | 0.01 | ECA22 |
|  | Hanoverian | 8 | 11 | 1.659 | 0 | 0 | 0.52 | ECA22 |
|  | Icelandic | 11 | 6 | 1.232 | 0 | 0 | 0.12 | ECA26 |
|  | Saddlebred | 6 | 15 | 1.036 | 0 | 0 | 0.8 | ECA10, 29, 7 |
| Gray (ECA25) | Andalusian | 6 | 10 | 1.534 | 0 | 0 | 0.12 | ECA11 |
|  | Arabian | 11 | 13 | 1.227 | 0 | 0 | 0.48 | ECA12 |
