# Supplementary material for: A High Density SNP Array for the Domestic Horse and Extant Perissodactyla: Utility for Association Mapping, Genetic Diversity, and Phylogeny Studies
Source: PLoS Genet. 2012 Jan 12;8(1):e1002451. doi: 10.1371/journal.pgen.1002451 (PMC3257288; doi:10.1371/journal.pgen.1002451)
Supplement: Table S15 — Summary of MC1R Haplotype Analysis on ECA3. Genotype data was phased as described in Materials and Methods and summary statistics on the chromosomes containing the associated haplotype are presented by breed. (DOCX) [file pgen.1002451.s024.docx]

**Table S15. Summary of *MC1R* Haplotype Analysis on ECA3.** Genotype data was phased as described in Materials and Methods and summary statistics on the chromosomes containing the associated haplotype are presented by breed.

|  |  | **Chromosome Attributes (Mb)** | | |  |
| --- | --- | --- | --- | --- | --- |
|  | **Chromosomes evaluated** | **Start** | **Stop** | **Length** | **#SNPs** |
| **Andalusian** | 7 | 35.897 | 36.977 | 1.080 | 16 |
| **Arabian** | 35 | 35.408 | 38.158 | 2.750 | 51 |
| **Belgian** | 48 | 35.408 | 36.977 | 1.569 | 23 |
| **Franches-Montagnes** | 19 | 35.408 | 36.977 | 1.569 | 23 |
| **French Trotter** | 19 | 34.001 | 38.158 | 4.157 | 88 |
| **Hanoverian** | 23 | 35.408 | 37.726 | 2.318 | 38 |
| **Icelandic** | 19 | 35.481 | 36.977 | 1.496 | 20 |
| **Mongolian** | 26 | 35.408 | 36.616 | 1.208 | 17 |
| **Norwegian Fjord** | 10 | 34.600 | 36.979 | 2.379 | 46 |
| **Quarter Horse** | 75 | 35.408 | 36.977 | 1.569 | 21 |
| **Saddlebred** | 41 | 35.408 | 37.74 | 2.332 | 39 |
| **Standardbred** | 10 | 34.599 | 38.157 | 3.558 | 75 |
| **Swiss Warmblood** | 27 | 35.408 | 37.8 | 2.392 | 41 |
| **Thoroughbred** | 41 | 35.640 | 38.158 | 2.518 | 48 |
| **Average** | 27.8 |  |  | 2.159 | 37.8 |
|  |  |  |  |  |  |
