# Supplementary material for: A High Density SNP Array for the Domestic Horse and Extant Perissodactyla: Utility for Association Mapping, Genetic Diversity, and Phylogeny Studies
Source: PLoS Genet. 2012 Jan 12;8(1):e1002451. doi: 10.1371/journal.pgen.1002451 (PMC3257288; doi:10.1371/journal.pgen.1002451)
Supplement: Text S1 — Supporting text includes additional information on: filtering of genotypes in Perissodactyla, the relationships between domestic horse breeds and between the domestic horse and the Przewalski's Horse, and genome wide association analysis. (DOCX) [file pgen.1002451.s025.docx]

**Supporting Text S1**

**A High Density SNP Array for the Domestic Horse and Extant Perissodactyla: Utility for Association Mapping, Genetic Diversity and Phylogeny Studies**

**Supporting Text**

**Filtering of genotypes in Perissodactyla: Table S8.** In the Hippomorpha filtering for genotyping intensity had little impact on genotyping rates, whereas filtering for signal intensity dramatically reduced the call rates in Ceratomorpha (Table S8). After filtering genotypes for signal intensity, mean individual genotyping rates in the Hippomorpha was 0.93, and three individuals were removed from further analysis due to low genotyping; one Przewalski’s Horse (genotyping rate 0.77), one Somali wild ass (genotyping rate 0.73) and one Kulan (genotyping rate 0.67). After filtering for signal intensity the individual genotyping rates in the Ceratomorpha were much lower, mean 0.12, and three individuals were removed for extremely low genotyping rates; one South African Black rhino (genotyping rate 0.002), and both Great Indian rhinos (genotyping rates 0.0003 and 0.0014).

**Relationships between domestic horse breeds and between the domestic horse and the Przewalski’s Horse: Figures S3 - S5.** MDS plots of pair-wise genetic distances in 6 dimensions were used to visualize the relationships between breeds. MDS plots of dimension one versus 2 separated horses into distinct breed groups, with less tight breed clustering in breeds with recent or ongoing admixture. Dimension 3 separated the Standardbred and the French Trotter, two breeds bred for a similar performance phenotype, from the other breeds. The Norwegian Fjord, Icelandic horse, Mongolian horse and the Belgian clustered together in the first three dimensions, but Belgian and Mongolian horses were separated from Icelandic and Fjord horses in dimension 4. Icelandic and Norwegian Fjord horses clustered tightly together in all 6 dimensions (data not shown). The Andalusian breed was not tightly clustered in dimension 6, suggesting possible population stratification within this breed.

**Genome Wide Association Analysis: Figures S6 – S8 and Tables S12 – S14.** Nine Mendelian coat color loci were genotyped in this set of 335 horses. Three of these traits had sufficient numbers of samples, either across all 14 breeds, or sometimes within a single breed, to attempt to map their known chromosomal locations. These were the recessive chestnut coat color locus on ECA3 due to a polymorphism in the *MC1R* gene [2] which abolishes eumelamin synthesis in body hair follicles; the recessive black coat color locus on ECA22 due to a polymorphism in the *ASIP* (agouti) [3] gene which results in uniform distribution of black pigment throughout the body; and the dominant gray locus on ECA25 caused by a 4.6 kb duplication within the *STX17* gene [4]. Since gray is epistatic to black and chestnut, and chestnut is epistatic to recessive black, a horse’s coat color phenotype for GWAS was determined in two ways: 1) by inference from genotypes at all 9 known coat color loci (see Materials and Methods); or 2) by inference only from the genotype at the locus of interest (*MC1R*, *ASIP*, or *STX17*) assuming a simple Mendelian trait.

The chestnut locus was easily mapped across breeds to ECA3 using a basic chi-square case-control allelic association analysis, regardless of how phenotype was inferred (Tables S12, and Figure S6). The black coat color locus was identified more clearly using the *ASIP* genotype without consideration of the other coat color loci (Tables S12 and Figure S7). The CMH test allowed both the chestnut and black coat color loci to be unambiguously mapped across breeds, with no false positives (Table S13). Gray was not mapped across all breeds using CMH, or structured association mapping using principal components or mixed-model analyses to control for underlying population structure (data not shown). Attempts to map the gray coat color locus across breeds by allelic association resulted in a large number of false positive associations, with the most significant p-values on ECA9 even after label-swapping permutations (Table S12 and Figure S8). The Cochran-Mantel- Haenszel (CMH) association test was also used due to elevated genomic inflation factors (lambda), high false discovery rates (Table S13), and visible inflation of p-values in the quantile-quantile (QQ) plots in the across breed chi square analysis (data not shown).

Within breed mapping of the coat color loci was not attempted unless there was a minimum of 6 cases and 6 controls (Table S14). The chestnut locus was successfully mapped in Quarter Horses (22 cases and 24 controls) and Thoroughbreds (11 cases and 26 controls). In the Saddlebred (13 cases and 18 controls) the lowest permuted p-values also assigned chestnut to the correct locus, but the locus did not reach genome-wide significance (defined as a permuted p-value < 0.05). The lowest permuted p-values did not assign chestnut to the correct locus in the Hanoverian (7 cases and 12 controls) or Swiss Warmblood (8 cases and 11 controls) (Table S14). A similar result was obtained when mapping the chestnut locus from the *MC1R* genotype alone, with the addition of successful mapping within the Arabian (14 cases and 10 controls). Fewer samples were available with which to attempt to map black or grey within breeds. However, the *ASIP* locus was successfully mapped in the Andalusian (6 cases and 10 controls), but not in several other breeds, when black was considered as a simple recessive trait. The gray phenotype was not successfully mapped within the two breeds attempted.

**References**

1. Vila C, Lenoard J, Beja-Pereira A (2006) Genetic Documentation of Horse and Donkey Domestication. In: Zeder M, Bradley D, Emshwiller E, Smith B, editors. Documenting Domestication: New Genetic and Archaeological Paradigms. Berkley: University of Claifornia Press. pp. 342-353.

2. Marklund L, Moller MJ, Sandberg K, Andersson L (1996) A missense mutation in the gene for melanocyte-stimulating hormone receptor (MC1R) is associated with the chestnut coat color in horses. Mamm Genome 7: 895-899.

3. Rieder S, Taourit S, Mariat D, Langlois B, Guerin G (2001) Mutations in the agouti (ASIP), the extension (MC1R), and the brown (TYRP1) loci and their association to coat color phenotypes in horses (Equus caballus). Mamm Genome 12: 450-455.

4. Rosengren PG, Golovko A, Sundstrom E, Curik I, Lennartsson J, Seltenhammer MH, Druml T, Binns M, Fitzsimmons C, Lindgren G, Sandberg K, Baumung R, Vetterlein M, Stromberg S, Grabherr M, Wade C, Lindblad-Toh K, Ponten F, Heldin CH, Solkner J, Andersson L (2008) A cis-acting regulatory mutation causes premature hair graying and susceptibility to melanoma in the horse. Nat Genet 40: 1004-1009.

**Supporting Figures**

**Figure S1.** Distance between informative SNPs across all 31 autosomes. Informative SNPs were defined as having MAF > 0.05 across all 14 breeds.

**Figure S2. Decline in genome-wide linkage disequilibrium across and within breeds.** Genome-wide linkage disequilibrium (LD) was estimated both within a given breed, and across all breeds, by calculating *r^2^* values between all pairs of SNPs with inter-SNP distances of less than 600 kb as described in Materials and Methods.

**Figure S3. Multidimensional scaling with 14 domestic horse breeds.** Metric multidimensional scaling analysis of pair-wise genetic distance was used as described in Materials and Methods to identify relationships between the 14 domestic horse breeds. In these plots dimensions 3 -6 (y axes) are always plotted against dimension 1 (x axis).

**Figure S4. Multidimensional scaling of Przewalski’s Horse and domestic horse breeds.** Metric multidimensional scaling analysis of pair-wise genetic distance was used as described in Materials and Methods to identify relationships between the 14 domestic breeds and the Przewalski’s Horse.

**Figure S5. Multidimensional scaling of Przewalski’s Horse and domestic horse breeds.** Metric multidimensional scaling analysis of pair-wise genetic distance was used as described in Materials and Methods to identify relationships between the 14 domestic breeds and the Przewalski’s Horse. In these plots dimensions 3- 6 (y axes) are plotted against dimension 1 (x axis).

**Figure S6. Mapping of the chestnut coat color locus across breeds based on phenotype inferred from all nine genotyped coat color alleles with known interactions (a), or based solely on *MC1R* genotype and chestnut as a simple recessive trait (b).** Phenotypes were inferred as described in Materials and Methods. Unstructured case-control association analyses using chi-square tests for allelic association were then performed on a pruned SNP set also as described in Materials and Methods. SNPs on each chromosome are labeled with a different color on the X axis as indicated. Also indicated on the X axis is the number of pruned SNPs across the genome included in the analysis.

**Figure S7. Mapping of the recessive black coat color locus across breeds based on phenotype inferred from all nine genotyped coat color alleles with known interactions (a), or based solely on *ASIP* genotype and black color as a simple trait (b).** Phenotypes were inferred as described in Materials and Methods. Unstructured case-control association analyses using chi-square tests for allelic association were then performed on a pruned SNP set also as described in Materials and Methods. SNPs on each chromosome are labeled with a different color on the X axis as indicated. Also indicated on the X axis is the number of pruned SNPs across the genome included in the analysis.

**Figure S8. Mapping of the gray coat color locus across breeds based on gray color as a simple dominant trait.** Gray phenotype was inferred as described in Materials and Methods. Corrected p-values after 10,000 label-swapping permutations are indicated. SNPs on each chromosome are labeled with a different color on the X axis as indicated. Also indicated on the X axis is the number of pruned SNPs across the genome included in the analysis.

**Figure S9. Conserved haplotype at the *MC1R* locus on ECA3.** For all breeds with the exception of the Standardbred, the length of minimal homozygosity for the chestnut allele is depicted. In the Standardbred, where no homozygotes were observed, the shortest length of the haplotype containing the chestnut allele is depicted. The number of chromosomes in each breed that contained the chestnut allele ranged from 7 in the Andalusian, and 10 in the Standardbred and Norwegian Fjord, to 41 in the Saddlebred and Thoroughbred, 48 in the Belgian and 75 in Quarter Horse, with an average of 27.8 across all 14 breeds. The number of SNPs on which the haplotype is based ranged from 16 in the Andalusian to 75 in the Standardbred, with an average of 37.8 across all 14 breeds. The length of the shared haplotype ranged from 1.08 Mb in Andalusian to 4.157 Mb in French Trotter, with an average length of 2.16 Mb across all 14 breeds. The complete list of chromosome numbers, numbers of SNPs on which the haplotype is based, and the length and coordinates of the shared haplotype are available in Table S13. The position of *MC1R* and the likely position of the centromere are depicted on the figure. Similar analyses resulted in no shared haplotypes for either *ASIP* or grey.

**Supporting Tables**

**Table S1. Validation rates for the assayed SNPs.** The number of selected SNPs from the seven discovery horses and Twilight that provided genotypes (converted), the number of validated SNPs in the equine sample set, and the validation fraction are indicated. SNPs with the highest validation rate were those originally ascertained in two breeds, especially if one of the horses was Twilight. SNPs with the lowest validation rate were originally ascertained in three breeds but were not present in Twilight’s sequence.

**Table S2. Breeds included in the study.** Breed, number of individuals, and geographic origin of horses genotyped in this study. *The control horses consisted of the 7 SNP discovery horses listed in Table S1 and the Thoroughbred mare Twilight used to generate the equine genome assembly.

**Table S3. Tests of Mendelian inheritance in trios.** Mendelian errors in each of the 18 nuclear trios were calculated and results are reported as Mendelian agreement as described in the Materials and Methods.

**Table S4. Inter-SNP spacing by chromosome.** Polymorphic SNPs were defined as having at least one heterozygous individual (*i.e.,* MAF > 0) across all 14 breeds.

**Table S5. Allele frequencies and genetic diversity in the domestic horse.** The mean and median MAF (including all SNPs), the number of polymorphic (MAF ≥ 0.01) and informative SNPs (MAF > 0.05), and genetic diversity as determined by heterozygosity (H_E_), are indicated and calculated as described in Materials and Methods.

**Table S6. Proportion of validated SNPs from each discovery breed across all breeds genotyped.** The impact of discovery breed on SNP validation rate across the genotyped sample set was determined as described in Materials and Methods.

**Table S7. Species, genotyping rates, sample and locus GC scores for unfiltered data in extant Perissodactyla.** Genotyping rates and GC scores reported using Illumina’s default calling parameters as described in Materials and Methods.

**Table S8. Quality scores, SNP conversion and SNP validation rates and mean heterozygosity for extant Perissodactyla after filtering for intensity and genotyping rate.** Genotyping rates, conversion and validation rates and observed hetrozygosities after filtering the data as described in Materials and Methods. Conversion and validation rates and heterozygosities are not reported for Ceratomorpha due to low genotyping quality.

**Table S9. Inbreeding coefficients in each breed.** Individual inbreeding coefficients (F) were estimated with 17,947 SNPs that were pruned for linkage equilibrium as described in Materials and Methods.

**Table S10. Mean pair-wise genetic distances in domestic horse populations.** Genetic distance (D) between pair-wise combinations of individuals was calculated as described in Materials and Methods.

**Table S11. Mean pair-wise distances between Przewalski’s Horse and domestic horse populations.** Genetic distance (D) between pair-wise combinations of individuals was calculated as described in Materials and Methods.

**Table S12. Chi square analysis for mapping of known coat color loci across the 14 breeds.** Chestnut, black and gray phenotypes were either inferred from the genotypes at 9 known coat color loci, or from the genotype of a single locus (designated as *MC1R* and *ASIP*) only, using known inheritance models as described in Materials and Methods. Case control association analyses were then performed on a pruned SNP set also as described in Materials and Methods. The genomic inflation factor lambda, the number of SNPs with an EMP2 < 0.05 after 10000 label-swapping permutations, the number of these SNPs within 5 Mb of the true locus (true positive SNPs), the length of the chromosomal segment at the true gene locus containing true positive SNPs, and the false discovery rate (percentage of all positive SNPs that are within 5 Mb of the true locus), are all indicated.

**Table S13. CMH analysis for mapping of known coat color loci across the 14 breeds.** Phenotypes were inferred from multi or single locus genotypes as described in Table 1 and the Materials and Methods. Stratified genome-wide association analysis was performed using the Cochran-Mantel-Haenszel (CMH) test. Horses were clustered for this test on the basis of the pair-wise population concordance test also as described in Materials and Methods. The genomic inflation factor lambda, the number of SNPs with an EMP2 < 0.05 after 10000 label-swapping permutations, the number of these SNPs within 5 Mb of the true locus (true positive SNPs), the length of the chromosomal segment at the true gene locus containing true positive SNPs, and the false discovery rate (percentage of all positive SNPs that are within 5 Mb of the true locus), are all indicated.

**Table S14. Chi-square tests of association for coat color loci within breeds.** Chestnut, black and gray phenotypes were either inferred from the genotypes at 8 known coat color loci, or from the genotype of a single locus (designated as *MC1R* and *ASIP*) only, using known inheritance models as described in Materials and Methods. Case-control association analyses were then performed on a pruned SNP set also as described in Materials and Methods. The genomic inflation factor lambda, the number of SNPs with an EMP2 < 0.05 after 10000 label-swapping permutations, the number of these SNPs within 5 Mb of the true locus, the lowest EMP2 value (if < 1.0), and the chromosomal location of the SNP with the lowest EMP2 value, are all indicated.

**Table S15. Summary of *MC1R* Haplotype Analysis on ECA3.** Genotype data was phased as described in Materials and Methods and summary statistics on the chromosomes containing the associated haplotype are presented by breed.
